# Novel 7-Chloro-(4-thioalkylquinoline) Derivatives: Synthesis and Antiproliferative Activity through Inducing Apoptosis and DNA/RNA Damage

**DOI:** 10.3390/ph15101234

**Published:** 2022-10-08

**Authors:** Joyce E. Gutiérrez, Esteban Fernandez-Moreira, Miguel A. Rodríguez, Michael R. Mijares, Juan Bautista De Sanctis, Soňa Gurská, Petr Džubák, Marián Hajdůch, Julia Bruno-Colmenarez, Luis Rojas, Denis Deffieux, Laurent Pouységu, Stéphane Quideau, Jaime Charris, Hegira Ramírez

**Affiliations:** 1 Laboratorio de Síntesis Orgánica, Facultad de Farmacia, Universidad Central de Venezuela, Los Chaguaramos 1041-A, Caracas 47206, Venezuela; 2 Escuela de Medicina UEES, Universidad Espíritu Santo, Samborondón 092301, Ecuador; 3 Unidad de Biotecnología, Facultad de Farmacia, Universidad Central de Venezuela, Los Chaguaramos 1041-A, Caracas 47206, Venezuela; 4 Instituto de Immunologia, Facultad de Medicina, Universidad Central de Venezuela, Los Chaguaramos 1050-A, Caracas 50109, Venezuela; 5 Institute of Molecular and Translational Medicine, Faculty of Medicine and Dentistry, Czech Advanced Technology and Research Institute, Palacky University, Hněvotínská 1333/5, 77900 Olomouc, Czech Republic; 6 Department of Chemical and Environmental Science, University of Limerick, V94 T9PX Limerick, Ireland; 7 Laboratorio de Productos Naturales, Facultad de Farmacia y Bioanálisis, Universidad de Los Andes, Merida 5101, Venezuela; 8 Institut des Sciences Moléculaires, Université de Bordeaux (UMR-CNRS 5255), 351 Cours de la Libération, CEDEX, 33405 Talence, France; 9 Institut Universitaire de France, CEDEX 05, 75231 Paris, France; 10 Dirección de Investigación, Universidad ECOTEC, Km. 13.5 Vía Samborondón, Guayaquil 092302, Ecuador

**Keywords:** antiproliferative activity, cell cycle, DNA/RNA damage, Sulfanyl-Sulfinyl-Sulfonyl groups, synthesis of 4-thioalkylquinoline

## Abstract

A series of 78 synthetic 7-chloro-(4-thioalkylquinoline) derivatives were investigated for cytotoxic activity against eight human cancer as well as 4 non-tumor cell lines. The results showed, with some exceptions, that sulfanyl **5**–**40** and sulfinyl **41**–**62** derivatives exhibited lower cytotoxicity for cancer cell lines than those of well-described sulfonyl N-oxide derivatives **63**–**82**. As for compound **81**, the most pronounced selectivity (compared against BJ and MRC-5 cells) was observed for human cancer cells from HCT116 (human colorectal cancer with wild-type p53) and HCT116p53−/− (human colorectal cancer with deleted p53), as well as leukemia cell lines (CCRF-CEM, CEM-DNR, K562, and K562-TAX), lung (A549), and osteosarcoma cells (U2OS). A good selectivity was also detected for compounds **73** and **74** for leukemic and colorectal (with and without p53 deletion) cancer cells (compared to MRC-5). At higher concentrations (5 × IC_50_) against the CCRF-CEM cancer cell line, we observe the accumulation of the cells in the G0/G1 cell phase, inhibition of DNA and RNA synthesis, and induction of apoptosis. In addition, X-ray data for compound **15** is being reported. These results provide useful scientific data for the development of 4-thioalkylquinoline derivatives as a new class of anticancer candidates.

## 1. Introduction

Cancer is a major public health problem as it is one of the leading causes of morbidity and mortality worldwide. An estimated 19.3 million new cancer cases and almost 10 million cancer deaths occurred in 2020 [1]. Current management of cancers is based on various principles ranging from chemotherapy to radiotherapy and also extends to surgical management depending on type and severity, as well as immunotherapy and combination therapy. Unfortunately, the most commonly used chemotherapeutic agents are accompanied by severe adverse effects, including limited bioavailability, toxicity, non-specificity, and promotion of recurrence or metastasis [2].

There are many efforts to reduce adverse effects during cancer therapy, such as preventing side effects on the nearby cells and tissues, increasing drug accumulation and efficacy in the lesion, and developing novel drug delivery and targeting systems [3]. The process of finding new therapeutic indications for currently used drugs, defined as ‘repurposing’, is receiving growing attention. Chloroquine (CQ) and hydroxychloroquine (HCQ), with an original indication to prevent or cure malaria, have been successfully used to treat several infections [4,5,6,7]. Among the biological effects of CQ and HCQ, it is important to highlight their antitumoral properties, likely due to their strong antiproliferative, antimutagenic, and inhibiting autophagy capacities. These effects make these drugs a possible option in the treatment of several tumors in association with radiotherapy and chemotherapy [8].

As a privileged fragment, quinoline is a rigid planar molecule, which is a pharmacophore present in the core of numerous physiologically active agents that display interesting therapeutic properties [9]. Structurally, quinoline can be readily modified with a broad range of substituent groups to provide the molecular diversity necessary to achieve a library of compounds, among which different members can show different biological effects [10,11,12,13,14,15,16,17]. Similarly, organic compounds displaying chalcogen atoms in their structure, such as sulfur, are well known and studies have demonstrated efficacious treatments with these types of compounds against disease models associated with β-hematin, adhesion, migration, invasion inhibition, apoptosis induction, oxidative stress, for their antimalarial and antitubercular actions, as hypocholesterolemic agents, and for their antiproliferative activity [14,18,19,20,21,22,23,24,25,26,27,28,29].

**Figure 1:**
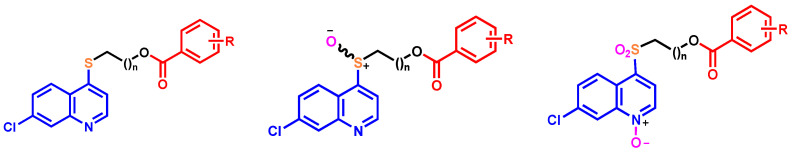
General structure of [(7-chloroquinolin-4-yl)sulfanyl, sulfinyl, and sulfonyl]alkyl benzoates.

Therefore, we designed and synthesized new molecules to further optimize CQ-based anti-cancer agents, in which we selectively modified the lateral sidechain of the 4-amino functionality with a sulfur-containing group and the incorporation of a variety of substituted carboxylic acids (Figure 1). We selected this approach because the 4-sulfanyl, sulfinyl, and sulfonyl-substituted CQ analogs have not yet been explored for anti-cancer activity. We have found that some of them are promising as they show more effective antiproliferative activities than CQ in a cancer-specific manner.

## 2. Results and Discussion

### 2.1. Chemistry

**Scheme 1:**
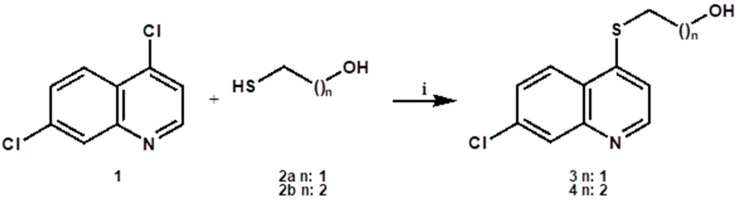
Synthesis of compounds **3**, **4**. Reagents and conditions. i: Et_3_N, dry EtOH, reflux, five days.

**Scheme 2:**
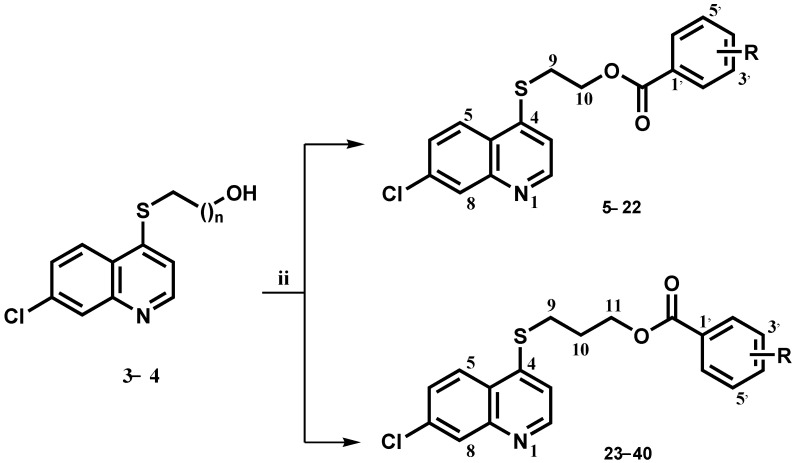
Synthesis of [(7-chloroquinolin-4-yl)sulfanyl]alkyl benzoate derivatives **5**–**40**. Reagents and conditions. ii: 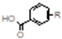, EDCI, DMAP, CH_2_Cl_2_, rt.

Based on our previous observations of the anticancer and antimalarial activity of 7-chloroquinoline derivatives [18,19,20,21,22], we chose to introduce diversity at position 4 by nucleophilic substitution of the chloride derivative **1** with different commercially available linear hydroxyalkylthiols **2** in the presence of an excess of triethylamine in ethanol under a variety of conditions involving different solvents (DMA, DMF, MeOH, EtOH, and THF), times (from 24 h until five days), and reaction temperatures (reflux) to generate compounds **3**, **4**. The best result was obtained using dry ethanol and a reflux temperature of 80 °C for 5 days, under a N_2_ atmosphere (Scheme 1). The yield of this reaction was moderate to good (52–65%). The chemoselectivity was not a problem despite the presence of a hydroxy (OH) group. The final compounds **5**–**40** were synthesized via a coupling reaction between **3** or **4** and a series of substituted benzoic acids, in the presence of N-(3-dimethylaminopropyl)-N′-ethylcarbodiimide hydrochloride (EDCI) and 4-(dimethylamino)-pyridine (DMAP) in CH_2_Cl_2_ for 24 h at room temperature (rt) [30]. The title compounds were isolated in good-to-excellent (58–99%) yields after purification by recrystallization or by column chromatography (Scheme 2).

NoRNoRNoRNoR
**5**
4-OMe
**14**
3,4,5-tri(OMe)
**23**
4-OMe
**32**
2-Cl
**6**
2,3-di(OMe)
**15**
2-Cl
**24**
2,3-di(OMe)
**33**
3-Cl
**7**
2,4-di(OMe)
**16**
3-Cl
**25**
2,4-di(OMe)
**34**
2,4-di(Cl)
**8**
2,5-di(OMe)
**17**
4-OMe-3-NO_2_
**26**
2,5-di(OMe)
**35**
4-OMe-3-NO_2_
**9**
2,6-di(OMe)
**18**
5-Me-2-NO_2_
**27**
3,5-di(OMe)
**36**
5-Me-2-NO_2_
**10**
3,4-di(OMe)
**19**
3,5-di(Me)
**28**
2,3,4-tri(OMe)
**37**
3,5-di(Me)
**11**
3,5-di(OMe)
**20**
4-CF_3_
**29**
2,4,5-tri(OMe)
**38**
4-CF_3_
**12**
2,3,4-tri(OMe)
**21**
4-C(Me)_3_
**30**
3,4,5-tri(OMe)
**39**
4-C(Me)_3_
**13**
2,4,5-tri(OMe)
**22**
2-OMe
**31**
2-OMe
**40**
2-F

**Scheme 3:**
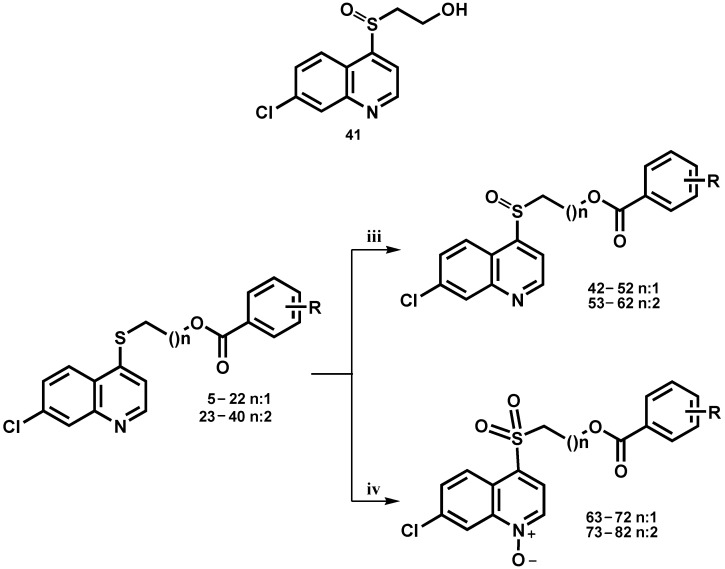
Synthesis of 2-[(7-chloroquinolin-4-yl)sulfinyl]ethyl or propyl benzoate **42**–**62** and 2-[(N-oxide 7-chloroquinolin-4-yl)sulfonyl]ethyl or propyl benzoate derivatives **63**–**82**. Reagents and conditions. **iii**: m-CPBA (1.2 mmol), CH_2_Cl_2_, 10 min, rt. **iv**: m-CPBA (2.5 mmol), CH_2_Cl_2_, 8–15 h, rt.

In addition, m-chloroperbenzoic acid (m-CPBA) was used as an oxidizing agent, while relying on different conditions of reaction such as times and equivalents of m-CPBA to prepare the sulfinyl derivatives **41**–**62** and the sulfonyl derivatives **63**–**82**. The title compounds were isolated in moderate-to-excellent (51–94%) and moderate-to-good (51–75%) yields, respectively, after purification by recrystallization or by column chromatography (Scheme 3).

NoRNoRNoRNoR
**42**
4-OMe
**53**
4-OMe
**64**
2,3-di(OMe)
**75**
2,5-di(OMe)
**43**
2,3-di(OMe)
**54**
2,3-di(OMe)
**65**
2,5-di(OMe)
**76**
2,4,5-tri(OMe)
**44**
2,5-di(OMe)
**55**
2,5-di(OMe)
**66**
2,4,5-tri(OMe)
**77**
3,4,5-tri(OMe)
**45**
2,4,5-tri(OMe)
**56**
2,4,5-tri(OMe)
**67**
3,4,5-tri(OMe)
**78**
2-OMe
**46**
3,4,5-tri(OMe)
**57**
3,4,5-tri(OMe)
**68**
2-OMe
**79**
4-OMe-3-NO_2_
**47**
2-OMe
**58**
2-OMe
**69**
4-OMe-3-NO_2_
**80**
5-Me-2-NO_2_
**48**
4-OMe-3-NO_2_
**59**
4-OMe-3-NO_2_
**70**
5-Me-2-NO_2_
**81**
3,5-di(Me)
**49**
5-Me-2-NO_2_
**60**
5-Me-2-NO_2_
**71**
3,5-di(Me)
**82**
4-CF_3_
**50**
3,5-di(Me)
**61**
3,5-di(Me)
**72**
4-CF_3_


**51**
4-CF_3_
**62**
4-CF_3_
**73**
4-OMe


**52**
4-C(Me)_3_
**63**
4-OMe
**74**
2,3-di(OMe)



In the ^1^H NMR spectra, the signal of the respective protons of each compound was checked based on their chemical shifts, multiplicities, and coupling constants. The aliphatic signals expected at upfield shifts were found from δH 1.70 to 4.60 ppm. The quinoline moiety protons appeared as a doublet around 6.5 ppm (d, J = 5 Hz) assigned to proton H3, a double doublet around 7.3 ppm (dd, J = 8 and 2 Hz) assigned to proton H6, a doublet around 7.5 ppm (d, J = 8 Hz) assigned to proton H5, a doublet around 7.9 ppm (d, J = 2 Hz) assigned to proton H8, and a doublet around 8.5 ppm (d, J = 5 Hz) assigned to proton H2. 

**Figure 2:**
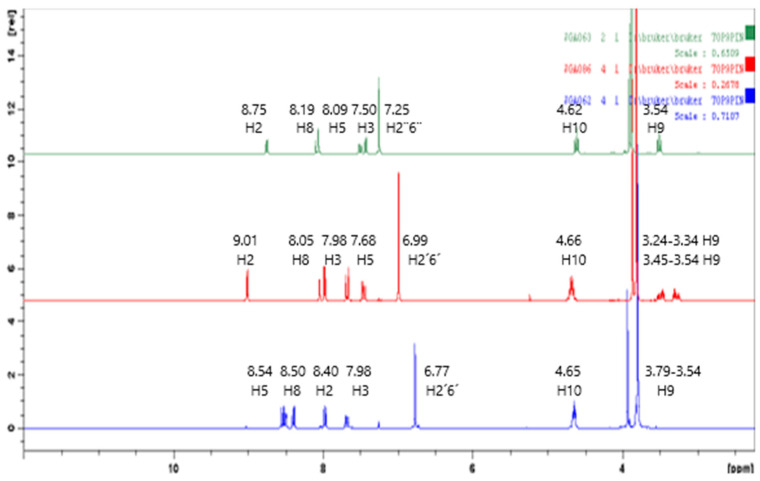
^1^H NMR spectra of compounds **14**, **46**, **67**.

**Figure 3:**
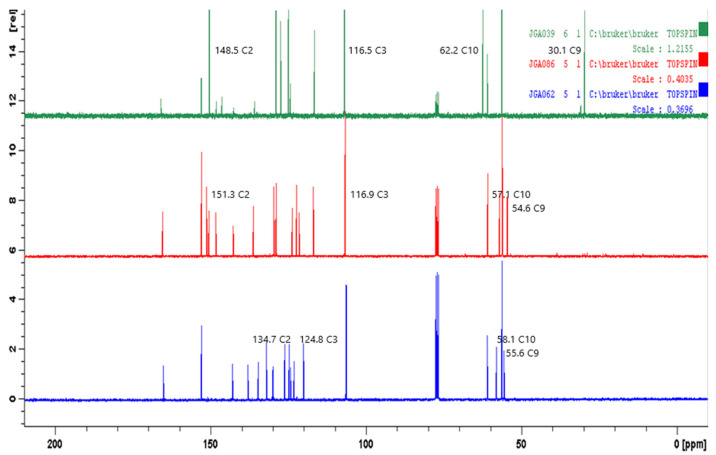
^13^C NMR spectra of compounds **14**, **46**, **67**.

**Figure 4:**
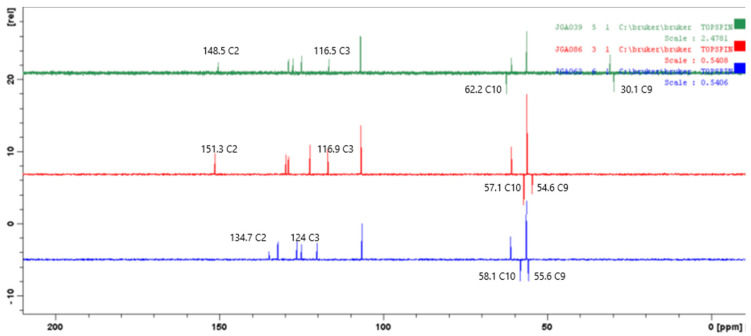
DEPT-135o spectra of compounds **14**, **46**, **67**.

However, when the sulfur atom was oxidized, significant changes were observed in the ^1^H NMR chemical shifts of both aliphatic and aromatic protons. For instance, for the 3,4,5-trimethoxy compounds **14**, **46**, and **67** (Figure 2): in compound **14**, the protons H9 appear as a doublet centered at 3.54 ppm with a coupling constant J = 6.9 Hz, whereas in compound **46**, protons H9, which are now prochiral, appear as two multiplets with δH values between 3.24 and 3.54 ppm; for compounds **67**, these protons H9 appear as a multiplet in the range of 3.79–3.83 ppm. For compounds **14** and **46**, a comparison of the ^1^H NMR chemical shifts of aromatic protons H3 and H5 revealed significant changes: the signal of proton H3 was de-shielded from 7.50 ppm in **14** (d, J = 4.9 Hz) to 7.98 ppm in **46** (d, J = 4.4 Hz), whereas the signal of proton H5 was shielded from 8.09 ppm in **14** (d, J = 8.9 Hz) to 7.68 ppm in **46** (d, J = 8.9 Hz). For compound **67**, oxidation of the nitrogen atom occurred in addition to the oxidation of the sulfur atom, thus prompting a significant change in the chemical shifts of all the protons of the quinoline core: proton H3 appeared at 7.98 ppm (d, J = 6.5 Hz), proton H2 resonated at 8.40 ppm (d, J = 6.5 Hz), and proton H5 was observed at 8.54 ppm (d, J = 9.1 Hz). Changes in the chemical shifts of the carbon atoms were also observed and confirmed by the ^13^C NMR and DEPT-135° spectra analysis (Figure 3 and Figure 4). For example, a significant difference was observed in the chemical shift of C9 in compound **14** δC 30.1 ppm, in comparison with δC at 54.6 and 55.6 ppm in compounds **46** and **67**, respectively, which could be associated with the inductive effect—I exerted by the SO and SO_2_ groups. In addition, a small difference is observed in the chemical shift of C10, which resonates at δC 62.2 ppm in compound **14**, as compared to the δC values of 57.1 and 58.1 ppm for **46** and **67**, respectively. On the other hand, a significant difference was observed in the chemical shift of C2 in compound **67** with δC 134.7 ppm, whereas it was 148.4 and 151.3 ppm in compounds **14** and **46**, respectively. A small difference in the chemical shift of C3 around 8 ppm was observed in compounds **14** and **46** (δC3 116.5 and 116.9 ppm, respectively) with respect to compound **67** (δC3 124.8 ppm). The aromatic region of the ^1^H NMR spectra featured signal patterns ranging from δH 6.5 to 8.0 ppm, which were characteristic of the substitution pattern of each aromatic ring. 

**Figure 5:**
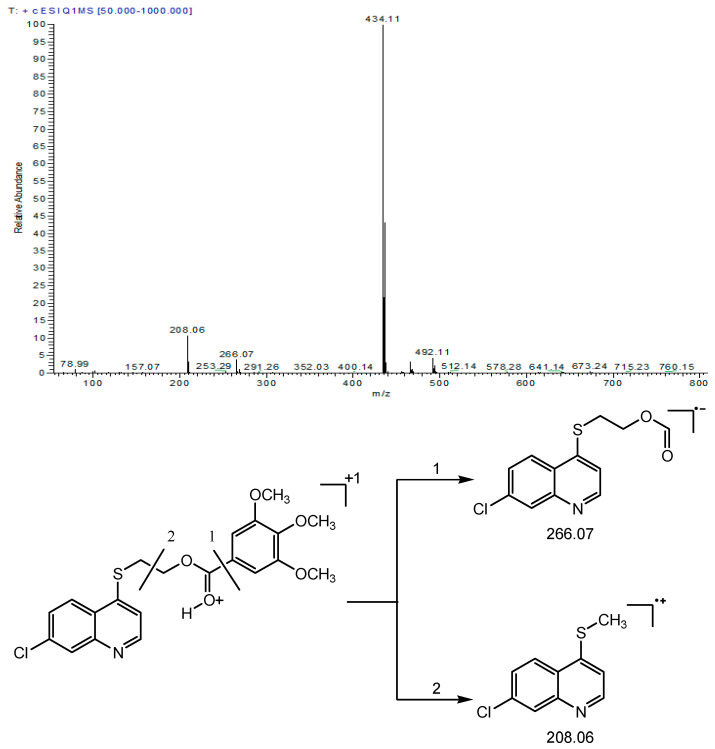
Mass spectrum of **14** and proposed fragmentation patterns.

**Figure 6:**
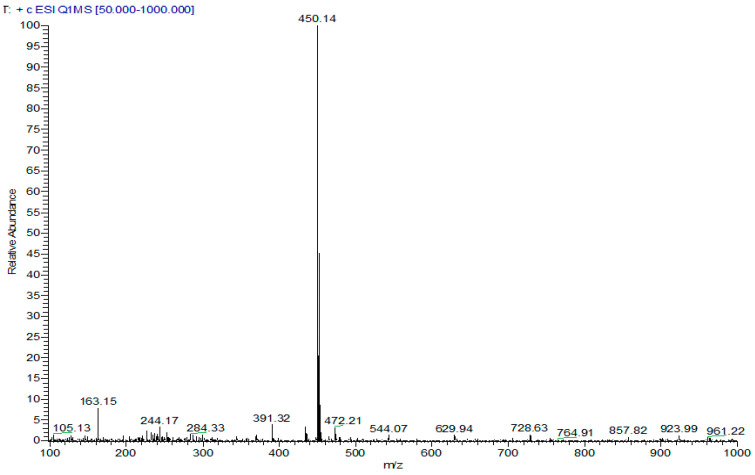

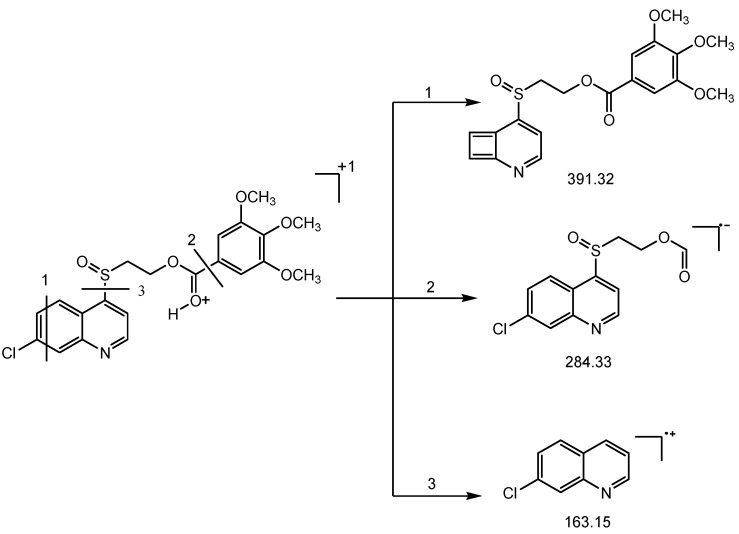
Mass spectrum of **46** and proposed fragmentation patterns.

**Figure 7:**
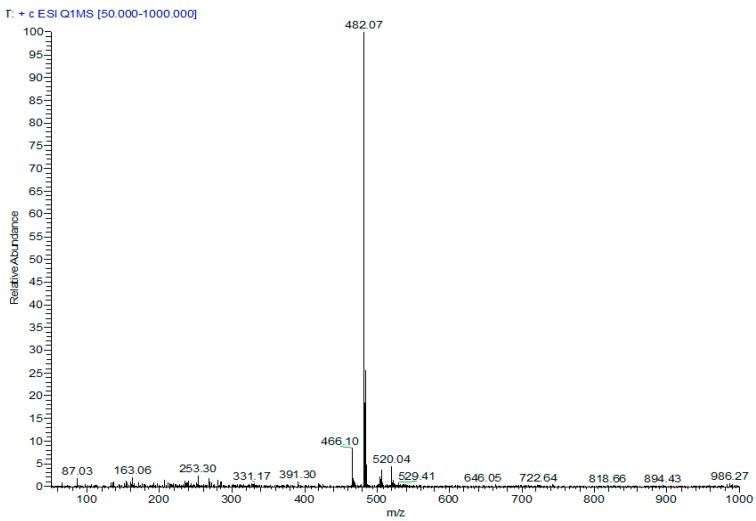

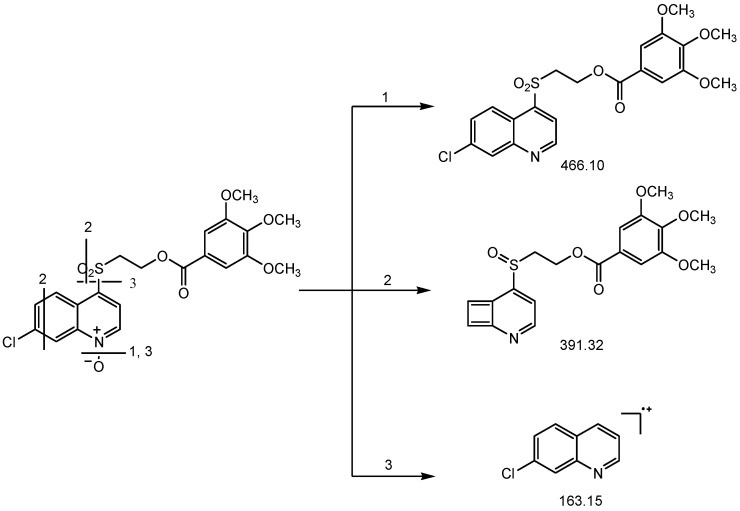
Mass spectrum of **67** and proposed fragmentation patterns.

Each product formation was further substantiated by its mass spectra. The EI-MS of representative compounds **14**, **46**, and **67** exhibited the molecular ion peak [M+H^+^] at *m*/*z* 434.11, 450.14, and 482.07 for C_21_H_20_ClNO_5_S, C_21_H_20_ClNO_6_S, and C_21_H_20_ClNO_8_S molecular formula, respectively (Figure 5, Figure 6 and Figure 7). The molecular ion of compound **14** undergoes multiple cleavages to give signals at *m*/*z* 266 and 208 (Figure 5). For compound **46**, the peak at *m*/*z* 391 was due to the elimination of H–C≡C–Cl from [M+] (Figure 6). For compound **67**, the peak at *m*/*z* 466 was due to an oxygen atom removal from [M], whereas the peak at *m*/*z* 391 could be explained by an oxygen atom elimination from the SO_2_ group followed by the elimination of H–C≡C–Cl from the peak at *m*/*z* 466 (Figure 7).

Crystal data, experimental parameters, and final refinement parameters for compound **15** are summarized in Table 1, and hydrogen bond geometries calculated with PLATON [31] are located in Table 2. Compound **15** crystallizes in the space group $P\bar{1}$ with cell parameters a = 7.9509 (7), b = 9.8444 (8), c = 11.6086 (10) Å; α = 81.312 (4), β = 89.355 (4), γ = 69.186 (4) (°); V = 838.70 (13) Å^3^, Z = 2, dc = 1.498 g cm^−^^3^. The moiety involves three rings of six members, all with planar conformation. Due to the geometry of the compound, intermolecular and classical hydrogen bonds are not observed in the structure. Only two intramolecular hydrogen bonds described by the atoms C10-H10, …, O1 and C14-H14, …, S1 are observed and these interactions are represented by the graph S(5). Analysis of interactions made with PLATON also evidences the presence of an interaction between the Cl1 atom and the electronic cloud of the ring composed of the C9-C10-C11-C12-C18-C17 atoms. At the same time, the packing is governed by π-π stacking between ring (1) composed of the atoms N1-C3-C4-C5-C16-C15 and ring (2) composed of the atoms C1-C2-C3-C4-C14-C13, and the distance between the centroids of both rings is 3.7997 (11) Å.

**Figure 8:**
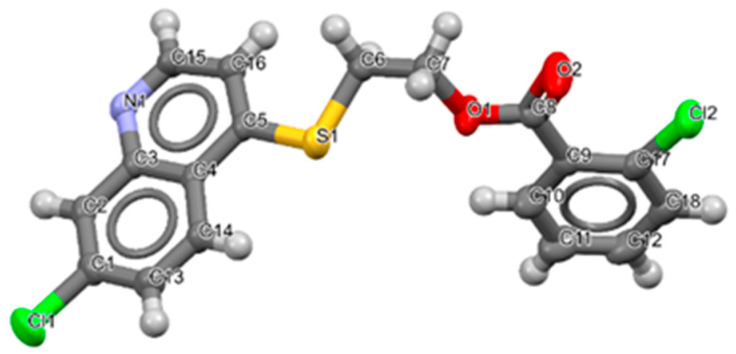
View of compound **15** with its atom-labeling scheme.

**Figure 9:**
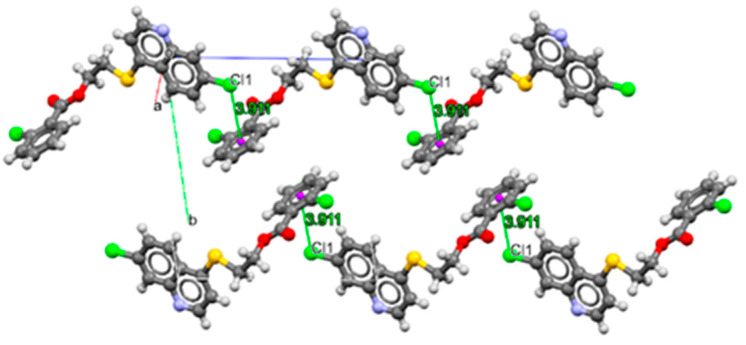
View of the C1-Cl1, …, Cg3 interactions.

The molecular structure plot with Mercury software [32] and its atom-labeling scheme are presented in (Figure 8), whereas (Figure 9) shows the Y-X, …, Cg interactions in the packing arrangement. 

### 2.2. Biological Activity

#### 2.2.1. Cytotoxicity

Chloroquine (CQ) and hydroxychloroquine (HCQ) are widely known drugs in the prevention or treatment of malaria, and they have lately been subjected to drug repurposing, since it has been reported that they also exhibit (i) activity against autoimmune diseases, arthritis, lupus, as immunomodulators, and (ii) great anticancer potential [4,5,6,7,8].

In vitro cytotoxic activity of final compounds **5**–**82** was evaluated after 3 days of incubation against eight cell lines derived from human solid tumors including lung (A549 cells) and colon (HCT116 and HCT116p53−/−) carcinomas, as well as leukemia cell lines (CCRF-CEM, CEM-DNR, K562, and K562-TAX) and osteosarcoma (U2OS cells). For comparison, non-malignant BJ and MRC-5 fibroblasts, and BJLD and MCR-5LD fibroblasts doxorubicin-resistant cell lines were used. Concentrations inhibiting the cell growth by 50% (IC_50_) were determined using a quantitative metabolic staining with 3-(4,5-dimethylthiazol-2yl)-5-(3-carboxymethoxyphenyl)-2-(4-sulfophenyl)-2H-tetrazolium (MTS) and are summarized in (Table 3) [33,34,35,36].

The results showed that the CCRF-CEM cell line was the most sensitive to tested 7-chloro-(4-thioalkylquinoline) derivatives **5**–**82**, particularly to **47**–**50**, **53**, **54**, **57**, **59**–**70**, and **72**–**82** (IC_50_ in the range of 0.55–2.74 µM) bearing sulfinyl and sulfonyl groups with a spacer between the quinoline core and the aromatic esters of two or three carbon atoms, which implies a correlation between the sulfur oxidation state, the spacer length, and the cytotoxic activity. With the exception of compounds **73**–**75** and **79**–**82** (IC_50_ in the range of 3.46–7.22 µM), the compounds were less active against its daunorubicin-resistant CEM-DNR counterparts.

Cytotoxic activity was determined by MTS assay following 72 h of incubation. IC_50_ is the lowest concentration that kills 50% of cells. The standard deviation in cytotoxicity assays is typically up to 20% of the average value. Compounds with IC_50_ > 50 µM are considered inactive: **5**–**40**, **42**–**46**, **51**, **52**, **55**, **56**, **58**, and **71**. The tested cell lines: A549 (lung adenocarcinoma), BJ (noncancerous human fibroblasts from foreskin), BJLD (human lung fibroblast doxorubicin-resistant), CCRF-CEM (childhood T cell acute lymphoblastic leukemia), a daunorubicin-resistant subline of CCRF-CEM cells (CEM-DNR bulk), HCT116 (colorectal carcinoma), HCT116p53−/− (HCT116 with deleted p53 gene), K562 (chronic myeloid leukemia), K562-TAX (chronic myeloid leukemia paclitaxel-resistant subline), MRC-5 (noncancerous human lung fibroblasts), MRC-5LD (noncancerous human lung fibroblasts doxorubicin-resistant), and U2OS (osteosarcoma).

With a few exceptions, in the case of K562 cells and the corresponding drug-resistant K562-TAX cell line, a more significant difference was observed for sulfinyl derivatives with a spacer of two or three atoms in the range of 1.1–2.0 times higher cytotoxicity against K562-TAX than K562 cell line. On the other hand, with the exception of **63**, **67**, **68**, **79**, **80**, and **82**, sulfonyl compounds with a spacer of two or atoms were in the range of 1.1–2.3 times more potent against K562 than the resistant K562-TAX cell line. These results indicate that other mechanisms than P-glycoprotein, which is common for both cell lines, are responsible for the resistance.

With the exception of sulfinyl derivatives **59**, **60** and sulfonyl derivatives with a spacer of three carbon atoms **73**–**75** and **77**–**82** (IC_50_ 5.32–9.02 µM), the rest of the compounds can be considered inactive against human lung adenocarcinoma A549 (IC_50_ > 10 µM).

Cytotoxic activity of all compounds tested against colon carcinoma (HCT116 and HCT116p53−/−) cell lines were similar. The most efficient were the sulfonyl N-oxide derivatives with a spacer of three carbon atoms **73**–**74** and **79**–**82** (IC_50_ 1.99–4.9 µM) against HCT116 cell line, whereas in the case of HCT116p53−/− the compounds were **73**, **74**, **79**, and **81** (IC_50_ 2.24, 3.23, 4.98, and 4.76 µM, respectively). A slight increase in cytotoxic activity was observed for compounds **59**, **60**, **63**, **73**–**75**, and **79**–**82** against human cancer cells from osteosarcoma (U2OS) (IC_50_ 4.95–5.81 µM).

In contrast, except for sulfonyl N-oxide derivatives with a spacer of two carbon atoms **63**–**69** (IC_50_ 2.02–8.41 µM), which were found toxic against BJLD and MRC-5LD cell lines, the rest of the compounds were less toxic on non-cancer cell lines BJ, MRC-5, BJLD, and MRC-5LD than the eight cancer cell lines evaluated: CCRF-CEM, CEM-DNR, K562, K562-TAX, A549, HCT116, HCT116p53−/−, and U2OS.

From these results, it is obvious, with some exception, that sulfanyl **5**–**40** and sulfinyl **41**–**62** derivatives exhibited lower cytotoxicities for cancer cell lines than that of well-described sulfonyl N-oxide derivatives **63**–**82**. As for compound **81**, the most pronounced selectivity (compared against BJ and MRC-5 cells) was observed for human cancer cells from HCT116 (human colorectal cancer with wild-type p53) and HCT116p53−/− (human colorectal cancer with deleted p53), as well as leukemia cell lines (CCRF-CEM, CEM-DNR, K562, and K562-TAX), lung (A549), and osteosarcoma cells (U2OS). A good selectivity was also detected for compounds **73** and **74** for leukemic and colorectal (with and without p53 deletion) cancer cells (compared to MRC-5). Concerning compound **79**, it exhibited a moderate cancer cell selectivity (compared to BJ and MRC-5 cell lines) for A549 cells, which was completed by a good selectivity (compared to BJ and MRC-5 cells) to cancer cells derived from lung, colon carcinoma, as well as to leukemic and osteosarcoma cells.

As expected, cytotoxicity of the tested compounds was significantly affected by the oxidation state of the sulfur atom attached at the C-4 position of the quinoline, by the oxidation of the quinolinic nitrogen, by the number of the carbon spacer, and by the type of substituent displayed at the benzoate moiety. Based on the data obtained in this study, we concluded that the derivatives with a higher oxidation state of the sulfur and nitrogen atoms, combined with the presence of methoxy, methyl, and nitro groups on their benzoate moiety, exhibited the highest cytotoxicity. 

#### 2.2.2. Cell Cycle and Cell Death Analysis

On the other hand, we wanted to find out whether compounds **48**–**50**, **53**, **54**, **57**, **59**–**63**, **65**–**70**, and **72** can stop the cell cycle of cancer cells, as it has been previously reported for chloroquine [37,38,39,40,41]. For a more detailed description of the biological activity of the studied derivatives, we performed a cell cycle analysis of the most sensitive CCRF-CEM cells after 24 h of incubation with the novel 7-chloro-(4-thioalkylquinoline) derivatives (Table 4).

The effect of compounds on cell cycle distribution was determined to gain insights into the mechanism of its antiproliferative activity. As can be seen in (Table 4), a 24 h exposure of CCRF-CEM cells to growth-suppressive concentrations of 7-chloro-(4-thioalkylquinoline) derivatives (1 × IC_50_ and 5 × IC_50_ μM) resulted in a significant accumulation of cells in G2/M phases, which was accompanied by an increase in cells with G0/G1, and a decrease in S, DNA, and RNA content. Except compound **70**, all tested compounds exhibited a dose-dependent increase in the population of mitotic (pH3Ser10 positive) cells. For example, as compared with control, the percentage of cells in G2/M phases was increased by treatment with 5 × IC_50_ μM of compounds for 24 h (Table 4). Compounds also induced distinct sub-G1 values, which represent the population of apoptotic and dead cells. As shown in (Table 4), there was a marked increase in the sub-G1 from 2.91 in untreated cells. 5-Bromo-2-deoxyuridine (BrDU) is incorporated into newly synthesized DNA, and 5-bromouridine (BrU) pulse labeling is therefore commonly used as a proliferation marker. Low BrDU and BrU incorporation into the DNA or RNA, respectively, of treated cells with all compounds at 5 × IC_50_ reflected inhibition of DNA and RNA synthesis, indicating irreversible apoptotic changes. The percentage of BrU negative cells incorporating 5-bromouridine is proportional to the transcriptional activity of CCRF-CEM cells.

These results suggested that these compounds could block the cell cycle and induce apoptosis and death in CCRF-CEM cells in a dose-dependent manner in vitro.

## 3. Materials and Methods

### 3.1. Chemistry

All chemicals and solvents were purchased from different chemical suppliers and were used without further purification unless stated otherwise. Dichloromethane (DCM) was distilled under nitrogen immediately before use. The drying agent used for DCM was calcium hydride. Reactions were monitored by thin layer chromatography (TLC) carried out on aluminum sheets precoated with silica gel 60 F254 (Merck KGaA, Darmstadt, Germany). Compounds were visualized under UV light (254 nm). Column chromatography was performed on Merck silica gel 60 (40–63 µm) as a stationary phase. Melting points were measured in open capillary tubes in a Thomas Hoover^TM^ apparatus (Thomas Scientific, Seattle, WA, United States) and are uncorrected. IR spectra were determined as KBr pellets on a Shimadzu^TM^ model 470 spectrophotometer (Shimadzu Co., Kyoto, Japan) and are expressed in cm^−1^. The ^1^H and ^13^C NMR spectra were recorded on a Bruker Avance^TM^ 300 (300 MHz/75.5 MHz) (Bruker Bioscience, Billerica, MA, United States) or a JEOL Eclipse^TM^ 270 (270 MHz/67.9 MHz) (JEOL Ltd., Tokyo, Japan) spectrometer using CDCl_3_ as the solvent, and are reported in ppm downfield from the residual CHCl_3_ (δ 7.25 ppm for ^1^H-NMR and 77.0 ppm for ^13^C-NMR). Signal multiplicity is given as singlet (s), doublet (d), double doublet (dd), multiplet (m), quartet (q), where coupling constant (J) values were estimated in Hertz. A Perkin Elmer^TM^ 2400 CHN elemental analyzer (Perkin Elmer, Inc., Waltham, MA, United States) was used to obtain the elemental analyses, and the results were within ±0.4% of the predicted values. Exact molecular masses were determined on a Finnigan TSQ Quantum Ultra (IET. Ltd., Mundelein, IL, USA) spectrometer equipped with an electrospray ion source. 

#### 3.1.1. General Procedure for the Synthesis of Compounds **3**,**4**

To a solution of 4,7-Dichloroquinoline **1** (5.0 g 25 mmol) in dry ethanol (100 mL) was added dropwise mercapto alcohol **2a** or **2b** (30 mmol) and triethylamine (5.3 mL, 37.5 mmol). The mixture was stirred at reflux temperature (80 °C) for 5 days, under a N_2_ atmosphere and then allowed to cool down to room temperature. The solvent was evaporated under reduced pressure. To the resulting solid was added ethyl acetate (150 mL), and the organic layer was subsequently washed with water (100 mL), with 10% sodium bicarbonate (2 × 20 mL) and a saturated NaCl solution (50 mL). Anhydrous sodium sulfate was finally added to the organic layer, which was then filtered and evaporated under reduced pressure. The compounds were then purified by column chromatography.

2-[(7-Chloroquinolin-4-yl)sulfanyl]ethanol (**3**). Column chromathography DCM:EtOAc:MeOH (7:2:1). White solid, yield: 52%; m.p. 106–107 °C, (161 °C) [27]; IR (KBr) cm^−^^1^: 3270, 2985, 1593; ^1^H NMR (CDCl_3_, 300 MHz) δ ppm: 3.33 (t, 2H, H9, J = 6.1 Hz), 3.99 (t, 2H, H10, J = 6.1 Hz), 7.21 (d, 1H, H3, J = 4.9 Hz), 7.48 (dd, 1H, H6, J = 2.0, 9.0 Hz), 8.04–8.07 (m, 2H, H5,8), 8.65 (d, 1H, H2, J = 4.8 Hz); ^13^C NMR (CDCl_3_, 75 MHz) δ ppm: 34.1 (C9), 60.2 (C10), 116.4 (C3), 124.9 (C5), 125,1, 127.4 (C8), 128.8 (C6), 135.8, 147.1, 147.9, 150.1 (C2). Anal. calcd. for: C_11_H_10_ClNOS: C 55.11, H 4.20, N 5.84; Found: C 55.10, H 4.23, N 5.97. MS: *m*/*z* 240.02 (M+H^+^. 11%).

3-[(7-Chloroquinolin-4-yl)sulfanyl]propan-1-ol (**4**). Column chromathography DCM:EtOAc:MeOH (7:2.5:0.5). Yellow solid, yield: 71%; m.p. 125–127 °C; IR (KBr) cm^−^^1^: 3450, 2850, 1598; ^1^H NMR (CDCl_3_, 300 MHz) δ ppm: 2.01–2.09 (m, 2H, H10), 2.48 (brs, 1H, OH), 3.23 (t, 2H, H9, J = 7.2 Hz), 3.86 (t, 2H, H11, J = 5.9 Hz), 7.15 (d, 1H, H3, J = 4.9 Hz), 7.46 (dd, 1H, H6, J = 2.2, 8.9 Hz), 8.01 (d, 1H, H5, J = 8.9 Hz), 8.03 (d, 1H, H8, J = 2.3 Hz), 8.63 (d, 1H, H2, J = 4.9 Hz). ^13^C NMR (CDCl_3_ 75 MHz) δ ppm: 27.7 (C10), 31.1 (C9), 60.9 (C11), 116.0 (C3), 125.1, 127.4, 128.8, 135.8, 147.9, 148.3, 150.2. Anal. calcd. for: C_12_H_12_ClNOS: C 56.80, H 4.77, N 5.52; Found: C 56.77, H 4.79, N 5.75. MS: *m*/*z* 254.04 (M+H^+^. 13%).

#### 3.1.2. General Procedure for the Synthesis of Compounds **5**–**40**

A solution of the selected benzoic acid derivative (1.2 mmol) in dry DCM (15 mL) was treated with EDCI (1.5 mmol) and DMAP (0.4 mmol). The mixture was shaken at −10 °C for 30 min. The respective intermediates, **3** or **4** (0.65 mmol), dissolved in dry DCM (1 mL), were slowly added, and the resulting mixture was left stirring for 24 h at room rt, under a N_2_ atmosphere. Next, water was added and the aqueous fraction was extracted with DCM (2 × 10 mL). The organic layer was washed with 10% sodium bicarbonate (2 × 10 mL), a saturated NaCl solution (3 × 10 mL), and finally dried over Na_2_SO_4_, filtered, and evaporated under reduced pressure to give the crude product. The compounds were then purified by recrystallization or column chromatography.

2-[(7-Chloroquinolin-4-yl)sulfanyl]ethyl-4-methoxybenzoate (**5**). Column chromathography DCM:EtOAc (8:2). White solid, yield: 92%; m.p. 116–118 °C; IR (KBr) cm^−^^1^: 3030, 2971, 1699, 1242; ^1^H NMR (CDCl_3_, 300 MHz) δ ppm: 3.48 (t, 2H, H9, J = 6.9 Hz), 3.85 (s, 3H, OMe), 4.58 (t, 2H, H10, J = 6.7 Hz), 6.89 (d, 2H, H3′,5′, J = 8.9 Hz), 7.41 (d, 1H, H3, J = 4.7 Hz), 7.50 (dd, 1H, H6, J = 2.3, 8.9 Hz), 7.95 (d, 2H, H2′,6′, J = 8.9 Hz), 8.06 (d, 1H, H8, J = 2.3 Hz), 8.10 (d, 1H, H5, J = 8.9 Hz), 8.72 (d, 1H, H2, J = 4.7 Hz). ^13^C NMR (CDCl_3_, 75 MHz) δ ppm: 30.0 (C9), 55.6 (OMe), 62.2 (C10), 113.7 (C3′ or 5′), 113.8 (C3′ or 5′), 116.7 (C3), 121.9, 125.1 (C5), 127.6 (C6), 128.8 (C8), 131.8 (C2′ or 6′), 132.2 (C2′ or 6′), 136.0, 147.0, 148.0, 150.4 (C2), 163.8, 166.2 (C11). Anal. calcd. for: C_19_H_16_ClNO_3_S: C 61.04, H 4.31, N 3.75; Found: C 60.98, H 4.33, N 3.87. MS: *m*/*z* 374.06 (M+H^+^. 22%).

2-[(7-Chloroquinolin-4-yl)sulfanyl]ethyl 2,3-dimethoxybenzoate (**6**). Column chromathography DCM:EtOAc (8:2). White solid, yield: 91%; m.p. 108–109 °C; IR (KBr) cm^−^^1^: 2941, 1700, 1258; ^1^H NMR (CDCl_3_, 270 MHz) δ ppm: 3.52 (t, 2H, H9, J = 6.7 Hz), 3.88 (s, 3H, OMe), 3.90 (s, 3H, OMe), 4.61 (t, 2H, H10, J = 6.7 Hz), 7.07 (d, 1H, H5′, J = 4.5 Hz), 7.25–7.28 (m, 2H, H3, 4′), 7.44 (d, 1H, H6′, J = 4.7 Hz), 7.52 (d, 1H, H6, J = 8.9 Hz), 8.08 (d, 1H, H5, J = 8.9 Hz), 8.15 (brs, 1H, H8), 8.72 (d, 1H, H2, J = 4.9 Hz). ^13^C NMR (CDCl_3_, 67.9 MHz) δ ppm: 29.8 (C10), 56.1 (OMe), 61.5 (OMe), 62.3 (C11),116.2 (C3 or 3′), 116.6 (C3 or 3′), 122.2 (C4′), 123.9 (C5′), 125.0 (C5), 125.3, 127.4 (C6), 128.9 (C8), 135.7, 146.4, 148.1, 149.3, 150.4 (C2), 153.6, 165.8 (C11). Anal. calcd. for: C_20_H_18_ClNO_4_S: C 59.48, H 4.49, N 3.47; Found: C 59.48, H 4.47, N 3.72. MS: *m*/*z* 404.07 (M+H^+^. 21%).

2-[(7-Chloroquinolin-4-yl)sulfanyl]ethyl 2,4-dimethoxybenzoate (**7**). White solid, yield: 86% crystallized from ethanol; m.p. 107–108 °C; IR (KBr) cm^−1^: 2944, 1678, 1273; ^1^H NMR (CDCl_3_, 270 MHz) δ ppm: 3.53 (t, 2H, H9, J = 6.9 Hz), 3.85 (s, 3H, OMe), 3.88 (s, 3H, OMe), 4.57 (t, 2H, H10, J = 6.9 Hz), 6.43–6.47 (m, 2H, H3′,5′), 7.54–7.57 (m, 2H, H3, 6), 7.79 (dd, 1H, H6′, J = 1.7, 7.7 Hz), 8.11 (d, 1H, H5, J = 9.2 Hz), 8.25 (d, 1H, H8, J = 1.9 Hz), 8.71 (d, 1H, H2, J = 5.2 Hz). ^13^C NMR (CDCl_3_, 67.9 MHz) δ ppm: 30.1 (C9), 55.6 (OMe), 56.1 (OMe), 61.7 (C10), 99.1 (C5′), 104.9 (C3′), 111.5, 116.6 (C3), 125.1 (C5), 127.4 (C8), 128.2 (C6), 133.9 (C6′), 137.1, 139.5, 148.2 (C2), 161.9, 164.9, 165.1 (C12). Anal. calcd. for: C_20_H_18_ClNO_4_S: C 59.48, H 4.49, N 3.47; Found: C 59.50, H 4.49, N 3.67. MS: *m*/*z* 404.07 (M+H^+^. 13%).

2-[(7-Chloroquinolin-4-yl)sulfanyl]ethyl-2,5-dimethoxybenzoate (**8**). Column chromathography DCM:EtOAc (8:2). White solid, yield: 99%; m.p. 98–100 °C; IR (KBr) cm^−^^1^: 3048, 2925, 1700, 1240; ^1^H NMR (CDCl_3_, 270 MHz) δ ppm: 3.56 (t, 2H, H9, J = 6.9 Hz), 3.76 (s, 3H, OMe), 3.84 (s, 3H, OMe), 4.61 (t, 2H, H10, J = 6.9 Hz), 6.92 (d, 1H, H3′, J = 9.2 Hz), 7.05 (dd, 1H, H4′, J = 3.2, 9.2 Hz), 7.28 (d, 1H, H3, J = 4.4 Hz), 7.57–7.60 (m, 2H, H6,6′), 8.12 (d, 1H, H5, J = 9.1 Hz), 8.33 (brs, 1H, H8), 8.72 (d, 1H, H2, J = 4.5 Hz). ^13^C NMR (CDCl_3_, 67.9 MHz) δ ppm: 30.2 (C9), 56.0 (OMe), 56.8 (OMe), 62.1 (C10), 114.1 (C3′), 116.3 (C6 or 6′), 116.5 (C3), 120.0 (C4′), 125.0, 125.1 (C5), 126.2 (C8), 128.8 (C6 or 6′), 138.0, 146.9 (C2), 153.2, 153.9, 165.4 C11). Anal. calcd. for: C_20_H_18_ClNO_4_S: C 59.48, H 4.49, N 3.47; Found: C 59.49, H 4.48, N 3.61. MS: *m*/*z* 404.07 (M+H^+^. 21%).

2-[(7-Chloroquinolin-4-yl)sulfanyl]ethyl 2,6-dimethoxybenzoate (**9**). White solid, yield: 76% crystallized from ethanol; m.p. 116–118 °C; IR (KBr) cm^−1^: 3084, 2960, 1728, 1251; 1H NMR (CDCl_3_, 270 MHz) δ ppm: 3.52 (t, 2H, H9, J = 6.9 Hz), 3.78 (s, 3H, OMe), 3.84 (s, 3H, OMe), 4.61 (t, 2H, H10, J = 6.9 Hz), 6.55 (d, 2H, H3′,5′, J = 8.4 Hz), 7.29 (d, 1H, H4′, J = 8.4 Hz), 7.52–7.56 (m, 2H, H3,6), 8.09 (d, 1H, H5, J = 8.9 Hz), 8.23 (m, 1H, H8), 8.70 (d, 1H, H2). ^13^C NMR (CDCl_3_, 67.9 MHz) δ ppm: 28.8 (C9), 56.1 (OMe), 62.4 (C10), 104.2 (C3′, 5′), 112.4, 116.3 (C3), 125.1 (C5), 127.0 (C8), 128.4 (C6), 131.6 (C4′), 137.5, 147.7 (C2), 157.6, 166.2 (C11). Anal. calcd. for: C_20_H_18_ClNO_4_S: C 59.48, H 4.49, N 3.47; Found: C 59.46, H 4.53, N 3.65. MS: *m*/*z* 404.07 (M+H^+^. 17%).

2-[(7-Chloroquinolin-4-yl)sulfanyl]ethyl 3,4-dimethoxybenzoate (**10**). White solid, yield: 79% crystallized from ethanol; m.p. 109–110 °C; IR (KBr) cm^−1^: 2987, 1699, 1240; ^1^H NMR (CDCl_3_, 270 MHz) δ ppm: 3.58 (t, 2H, H9, J = 6.9 Hz), 3.90 (s, 3H, OMe), 3.93 (s, 3H, OMe), 4.62 (t, 2H, H10, J = 6.9 Hz), 6.86 (d, 1H, H5′, J = 8.7 Hz), 7.49 (d, 1H, H2′, J = 1.9 Hz), 7.59–7.62 (m, 3H, H3,6,6′), 8.12 (d, 1H, H5, J = 9.2 Hz), 8.38 (brs, 1H, H8), 8.74 (d, 1H, H2, J = 5.4 Hz). ^13^C NMR (CDCl_3_, 67.9 MHz) δ ppm: 30.4 (C9), 56.2 (OMe), 61.7 (C10), 110.6 (C5′), 112.4 (C2′), 114.7, 115.8 (C3), 121.7, 123.9 (C6′), 124.9, 125.1 (C5), 129.4 (C8), 138.9, 142.0, 145.3 (C2), 149.0, 153.9, 166.1 (C11). Anal. calcd. for: C_20_H_18_ClNO_4_S: C 59.48, H 4.49, N 3.47; Found: C 59.48, H 4.52, N 3.56. MS: *m*/*z* 404.07 (M+H^+^. 26%).

2-[(7-Chloroquinolin-4-yl)sulfanyl]ethyl 3,5-dimethoxybenzoate (**11**). White solid, yield: 84% crystallized from ethanol; m.p. 100–102 °C; IR (KBr) cm^−^^1^: 2993, 1718, 1240; ^1^H NMR (CDCl_3_, 270 MHz) δ ppm: 3.56 (t, 2H, H9, J = 6.9 Hz), 3.80 (s, 6H, OMe), 4.63 (t, 2H, H10, J = 6.7 Hz), 6.64 (t, 1H, H4′, J = 2.2 Hz), 7.12 (d, 2H, H2′, 6′, J = 2.2 Hz), 7.41 (d, 1H, H3, J = 4.9 Hz), 7.49 (dd, 1H, H6, J = 2.2, 9.1 Hz), 8.06 (d, 1H, H5, J = 8.9 Hz), 8.07 (d, 1H, H8, J = 1.9 Hz), 8.73 (d, 1H, H2, J = 5.2 Hz). ^13^C NMR (CDCl_3_, 67.9 MHz) δ ppm: 30.1 (C9), 55.6 (OMe), 62.6 (C10), 106.1 (C4′), 107.5 (C2′,6′), 116.9 (C3), 125.1 (C5 or 8), 125.3, 127.7 (C6), 128.8 (C5 or 8), 131.4, 136.2, 147.0, 147.8, 150.0 (C2), 160.9, 166.1 (C11). Anal. calcd. for: C_20_H_18_ClNO_4_S: C 59.48, H 4.49, N 3.47; Found: C 59.47, H 4.50, N 3.68. MS: *m*/*z* 404.07 (M+H^+^. 35%).

2-[(7-Chloroquinolin-4-yl)sulfanyl]ethyl 2,3,4-trimethoxybenzoate (**12**). White solid, yield: 80% crystallized from ethanol; m.p. 94–96 °C; IR (KBr) cm^−^^1^: 2980, 1699, 1245; ^1^H NMR (CDCl_3_, 270 MHz) δ ppm: 3.55 (t, 2H, H9, J = 6.7 Hz), 3.85 (s, 3H, OMe), 3.90 (s, 3H, OMe), 3.93 (s, 3H, OMe), 4.60 (t, 2H, H10, J = 6.7 Hz), 6.66 (d, 1H, H5′, J = 8.9 Hz), 7.52–7.56 (m, 3H, H3,6,6′), 8.09 (d, 1H, H5, J = 8.9), 8.26–8.31 (m, 1H, H8), 8.72 (d, 1H, H2, J = 5.2 Hz). ^13^C NMR (CDCl_3_, 67.9 MHz) δ ppm: 30.3 (C9), 56.2 (OMe), 61.0 (OMe), 61.8 (OMe), 61.9 (C10), 107.1 (C5′), 116.3 (C3), 117.0, 125.1 (C5), 126.9 (C6), 127.0 (C8), 128.5 (C6′), 137.6, 143.4, 144.7, 147.6 (C2), 155.0, 157.8, 165.1 (C11). Anal. calcd. for: C_21_H_20_ClNO_5_S: C 58.13, H 4.65, N 3.23; Found: C 58.13, H 4.67, N 3.40. MS: *m*/*z* 434.08 (M+H^+^. 75%).

2-[(7-Chloroquinolin-4-yl)sulfanyl]ethyl 2,4,5-trimethoxybenzoate (**13**). Column chromathography DCM:EtOAc (8:2). White solid, yield: 80%; m.p. 134–135 °C; IR (KBr) cm^−^^1^: 2930, 1666, 1204; ^1^H NMR (CDCl_3_, 300 MHz) δ ppm: 3.51 (t, 2H, H9, J = 7.1 Hz), 3.84 (s, 3H, OMe), 3.91 (s, 3H, OMe), 3.95 (s, 3H, OMe), 4.59 (t, 2H, H10, J = 7.0 Hz), 6.53 (s, 1H, H3′), 7.38 (s, 1H, H6′), 7.46 (d, 1H, H3, J = 4.9 Hz), 7.50 (dd, 1H, H6, J = 2.1, 8.9 Hz), 8.06 (d, 1H, H8, J = 2.3 Hz), 8.09 (d, 1H, H5, J = 9.2 Hz), 8.75 (d, 1H, H2, J = 4.9 Hz). ^13^C NMR (CDCl_3_, 75 MHz) δ ppm: 29.7 (C9), 56.1 (OMe), 56.5 (OMe), 57.0 (OMe), 62.0 (C10), 97.5 (C3′), 109.6, 114.5 (C6′), 116.6 (C3), 125.0 (C5), 125.1, 127.4 (C6), 129.0 (C8), 135.7, 142.6, 146.6, 148.1, 150.5 (C2), 154.2, 156.2, 165.2 (C11). Anal. calcd. for: C_21_H_20_ClNO_5_S: C 58.13, H 4.65, N 3.23; Found: C 58.15, H 4.64, N 3.27. MS: *m*/*z* 434.08 (M+H^+^. 83%).

2-[(7-Chloroquinolin-4-yl)sulfanyl]ethyl 3,4,5-trimethoxybenzoate (**14**). Column chromathography DCM:EtOAc (9:1). White solid, yield: 89%; m.p. 139–140 °C; IR (KBr) cm^−^^1^: 2892, 1700, 1209; ^1^H NMR (CDCl_3_, 270 MHz) δ ppm: 3.54 (d, 2H, H9, J = 6.9 Hz), 3.87 (s, 6H, OMe), 3.90 (s, 3H, OMe), 4.62 (t, 2H, H10, J = 6.9 Hz), 7.25 (s, 2H, H2′,6′), 7.50 (d, 1H, H3, J = 4.9 Hz), 7.54 (dd, 1H, H6, J = 1.9, 9.2 Hz), 8.09 (d, 1H, H5, J = 8.9 Hz), 8.19 (d, 1H, H8, J = 1.9 Hz), 8.75 (d, 1H, H2, J = 5.2 Hz). ^13^C NMR (CDCl_3_, 67.9 MHz) δ ppm: 30.1 (C9), 56.4 (OMe × 2), 61.0 (OMe), 62.2 (C10), 107.4 (C2′,6′), 116.5 (C3), 124.3, 125.1 (C5), 127.5 (C6), 128.3 (C8), 137.1, 143.3, 148.4 (C2), 148.5, 153.2, 166.0 (C11). Anal. calcd. for: C_21_H_20_ClNO_5_S: C 58.13, H 4.65, N 3.23; Found: C 58.13, H 4.65, N 3.34. MS: *m*/*z* 434.11 (M+H^+^. 100%).

2-[(7-Chloroquinolin-4-yl)sulfanyl]ethyl 2-chlorobenzoate (**15**). White solid, yield: 75% crystallized from ethanol; m.p. 108 °C; IR (KBr) cm^−^^1^: 2944, 1692, 1213; ^1^H NMR (CDCl_3_, 270 MHz) δ ppm: 3.55 (d, 2H, H9, J = 6.7 Hz), 4.65 (t, 2H, H10, J = 6.7 Hz), 7.27 (d, 1H, H3, J = 5.9 Hz), 7.29–7.32 (m, 1H, H5′), 7.40–7.50 (m, 2H, H3′,4′), 7.55 (dd, 1H, H6, J = 2.0, 8.9 Hz), 7.77 (d, 1H, H6′, J = 7.2 Hz), 8.11 (d, 1H, H5, J = 9.2 Hz), 8.20 (m, 1H, H8), 8.73 (s, 1H, H2). ^13^C NMR (CDCl_3_, 67.9 MHz) δ ppm: 30.1 (C9), 62.7 (C10), 116.6 (C3), 125.1 (C5), 126.7 (C5′), 127.8 (C8), 128.2 (C6), 129.4, 131.3 (C3′ or 4′), 131.5 (C6′), 133.1 (C3′ or 4′), 134.0, 137.0, 146.0, 148.7 (C2), 165.3 (C11). Anal. calcd. for: C_18_H_13_Cl_2_NO_2_S: C 57.15, H 3.46, N 3.70; Found: C 57.17, H 3.47, N 3.83. MS: *m*/*z* 378.10 (M+H^+^. 52%).

2-[(7-Chloroquinolin-4-yl)sulfanyl]ethyl 3-chlorobenzoate (**16**). White solid, yield: 79% crystallized from ethanol; m.p. 94–96 °C; IR (KBr) cm^−^^1^: 2937, 1730, 1246; ^1^H NMR (CDCl_3_, 270 MHz) δ ppm: 3.55 (d, 2H, H9, J = 6.7 Hz), 4.65 (t, 2H, H10, J = 6.7 Hz), 7.37 (t, 1H, H5′, J = 7.9 Hz), 7.49 (d, 1H, H3, J = 5.2 Hz), 7.53–7.55 (m, 1H, H4′), 7.58 (d, 1H, H6, J = 1.9 Hz), 7.86 (d, 1H, H6′, J = 7.6 Hz), 7.95 (t, 1H, H2′, J = 1.7 Hz), 8.11 (d, 1H, H5, J = 9.2 Hz), 8.25 (d, 1H, H8, J = 1.5 Hz), 8.74 (d, 1H, H2, J = 5.2 Hz). ^13^C NMR (CDCl_3_, 67.9 MHz) δ ppm: 30.3 (C9), 62.5 (C10), 116.5 (C3), 125.1 (C5), 126.9 (C8), 127.8 (C6′), 128.6 (C6), 129.8 (C2′), 129.9 (C5′), 131.2, 133.5 (C4′), 134.8, 147.8 (C2), 165.1 (C11). Anal. calcd. for: C_18_H_13_Cl_2_NO_2_S: C 57.15, H 3.46, N 3.70; Found: C 57.15, H 3.48, N 3.79. MS: *m*/*z* 378.09 (M+H^+^. 61%).

2-[(7-Chloroquinolin-4-yl)sulfanyl]ethyl 4-methoxy-3-nitrobenzoate (**17**). Column chromathography DCM:EtOAc (8:2). White solid, yield: 93%; m.p. 171–173 °C; IR (KBr) cm^−^^1^: 3030, 2963, 1717, 1519, 1233; ^1^H NMR (CDCl_3_, 300 MHz) δ ppm: 3.51 (t, 2H, H9, J = 6.8 Hz), 4.03 (s, 3H, OMe), 4.64 (t, 2H, H10, J = 6.8 Hz), 7.11 (d, 1H, H5′, J = 8.9 Hz), 7.39 (d, 1H, H3, J = 4.8 Hz), 7.50 (dd, 1H, H6′, J = 2.2, 8.9 Hz), 8.05 (d, 1H, H2′, J = 2.1 Hz), 8.09 (d, 1H, H5, J = 9.0 Hz), 8.13 (dd, 1H, H6, J = 2.2, 8.8 Hz), 8.45 (d, 1H, H8, J = 2.1 Hz), 8.76 (d, 1H, H2, J = 4.8 Hz). ^13^C NMR (CDCl_3_, 75 MHz) δ ppm: 30.0 (C9), 57.0 (OMe), 62.9 (C10), 113.3 (C5′), 116.9 (C3), 121.9, 125.1 (C5), 125.2, 127.4 (C6 or 2′), 127.6 (C6 or 2′), 129.1 (C8), 135.4 (C6′), 135.9, 146.2, 148.2, 150.4 (C2), 156.5, 164.2 (C11). Anal. calcd. for: C_19_H_15_ClN_2_O_5_S: C 54.48, H 3.61, N 6.69; Found: C 54.50, H 3.60, N 6.81. MS: *m*/*z* 419.07 (M+H^+^. 36%).

2-[(7-Chloroquinolin-4-yl)sulfanyl]ethyl 5-methyl-2-nitrobenzoate (**18**). Column chromathography DCM:EtOAc (8:2). White solid, yield: 79%; m.p. 93–95 °C; IR (KBr) cm^−^^1^: 3076, 2977, 1731, 1524, 1200; ^1^H NMR (CDCl_3_, 300 MHz) δ ppm: 2.46 (s, 3H, CH_3_), 3.49 (t, 2H, H9, J = 7.1 Hz), 4.61 (t, 2H, H10, J = 6.9 Hz), 7.37 (d, 1H, H3, J = 4.8 Hz), 7.39–7.43 (m, 2H, H4′,6′), 7.50 (dd, 1H, H6, J = 2.2, 8.9 Hz), 7.89 (d, 1H, H3′, J = 8.3 Hz), 8.05 (d, 1H, H8, J = 2.1 Hz), 8.08 (d, 1H, H5, J = 9.0 Hz), 8.74 (d, 1H, H2, J = 4.8 Hz). ^13^C NMR (CDCl_3_, 75 MHz) δ ppm: 21.4 (CH3), 29.1 (C9), 63.6 (C10), 116.8 (C3), 124.3 (C3′), 125.1 (C5), 125.2, 127.5 (C6), 127.7, 129.0 (C8), 130.0 (C6′), 132.2 (C4′), 135.9, 145.0, 145.5, 146.1, 148.2, 150.5 (C2), 165.8 (C11). Anal. calcd. for: C_19_H_15_ClN_2_O_4_S: C 56.65, H 3.75, N 6.95; Found: C 56.67, H 3.74, N 7.19. MS: *m*/*z* 403.15 (M+H^+^. 78%).

2-[(7-Chloroquinolin-4-yl)sulfanyl]ethyl 3,5-dimethylbenzoate (**19**). Column chromathography DCM:EtOAc (9:1). White solid, yield: 91%; m.p. 102–104 °C; IR (KBr) cm^−1^: 3024, 2972, 1729, 1204; ^1^H NMR (CDCl_3_, 300 MHz) δ ppm: 2.34 (s, 6H, Me), 3.49 (t, 2H, H9, J = 6.8 Hz), 4.60 (t, 2H, H10, J = 6.8 Hz), 7.19 (brs, 1H, H3), 7.42 (d, 1H, H4′, J = 4.7 Hz), 7.49 (d, 1H, H6, J = 8.9 Hz), 7.58 (brs, 2H, H2′,6′), 8.05–8.09 (m, 2H, H5,8), 8.74 (d, 1H, H2, J = 4.8 Hz). ^13^C NMR (CDCl_3_, 75 MHz) δ ppm: 21.3 (2 × CH3), 30.0 (C9), 62.5 (C10), 116.9 (C3), 125.2 (C5), 125.3, 127,4 (C2′,6′), 127.5 (C6), 129.1 (C8), 129.4, 135.1 (C4′), 135.9, 138.3, 146.6, 148.3, 150.5 (C2), 166.8 (C11). C_20_H_18_ClNO_2_S: C 64.59, H 4.88, N 3.77; Found: C 64.60, H 4.89, N 3.89. MS: *m*/*z* 372.12. (M+H^+^. 47%).

2-[(7-Chloroquinolin-4-yl)sulfanyl]ethyl 4-(trifluoromethyl)benzoate (**20**). Column chromathography DCM:EtOAc (8:2). White solid, yield: 75%; m.p. 134–136 °C; IR (KBr) cm^−^^1^: 3066, 2981, 1711, 1282; ^1^H NMR (CDCl_3_, 300 MHz) δ ppm: 3.51 (t, 2H, H9, J = 6.8 Hz), 4.65 (t, 2H, H10, J = 6,8 Hz), 7.37 (d, 1H, H3, J = 4.8 Hz), 7.48 (dd, 1H, H6, J = 2.3, 8.9 Hz), 7.69 (d, 2H, H3′,5′, J = 8.2 Hz), 8.04–8.09 (m, 4H, H5,8,2′,6′), 8.74 (d, 1H, H2, J = 4.8 Hz). ^13^C NMR (CDCl_3_, 75 MHz) δ ppm: 30.0 (C9), 63.0 (C10), 116.9 (C3), 125.1 (C5), 125.2 (C6), 125.6 (C3′,5′), 127.6 (C8), 129.1 (C2′,6′), 130.2, 132.8, 134.7, 135.1, 136.0, 146.2, 148.2, 150.5 (C2), 165.2 (C11). C_19_H_13_ClF_3_NO_2_S: C 55.41, H 3.18, N 3.40; Found: C 55.43, H 3.20, N 3.61. MS: *m*/*z* 412.16. (M+H+. 56%).

2-[(7-Chloroquinolin-4-yl)sulfanyl]ethyl-4-tert-butylbenzoate (**21**). Column chromathography DCM:EtOAc (9.5:0.5). White solid, yield: 91%; m.p. 120–122 °C; IR (KBr) cm^−1^: 3037, 2963, 1713, 1268; ^1^H NMR (CDCl_3_, 270 MHz) δ ppm: 1.32 (s, 9H, Me), 3.49 (t, 2H, H9, J = 6.9 Hz), 4.60 (t, 2H, H10, J = 6.9 Hz), 7.40–7.45 (m, 3H, H3,3′,5′), 7.49 (dd, 1H, H6, J = 2.2, 8.9Hz), 7.90 (dt, 2H, H2′,6′, J = 1.7, 6.9 Hz), 8.04 (d, 1H, H8, J = 2.0 Hz), 8.07 (d, 1H, H5, J = 9.2 Hz), 8.75 (d, 1H, H2, J = 4.9 Hz). ^13^C NMR (CDCl_3_, 67.9 MHz) δ ppm: 30.3 (C9), 31.1 (3 × CH3), 35.2, 62.3 (C10), 117.1 (C3), 125.2, 125.3 (C5), 125.5 (C3′,5′), 126.8, 127.5 (C6), 129.0 (C8), 129.6 (C2′,6′), 135.9, 146.6, 148.2, 150.4 (C2), 157.2, 166.4 (C11). C_22_H_22_ClNO_2_S: C 66.07, H 5.54, N 3.50; Found: C 66.05, H 5.56, N 3.74. MS: *m*/*z* 400.13. (M+H^+^. 33%).

2-[(7-Chloroquinolin-4-yl)sulfanyl]ethyl-2-methoxybenzoate (**22**). Column chromathography DCM:EtOAc (8:2). White solid, yield: 87%; m.p. 98–99 °C; IR (KBr) cm^−1^: 3020, 2980, 1715, 1220; ^1^H NMR (CDCl_3_, 300 MHz) δ ppm: 3.48 (t, 2H, H9, J = 6.9 Hz), 3.89 (s, 3H, OMe), 4.59 (t, 2H, H10, J = 6.9 Hz), 6.92–6.98 (m, 2H, H3′,5′), 7.41 (d, 1H, H3, J = 4.8 Hz), 7.45–7.50 (m, 2H, H6,4′), 7.76 (dd, 1H, H6′, J = 1.4, 7.7 Hz), 8.04–8.08 (m, 2H, H5,8), 8.72 (d, 1H, H2, J = 4.8 Hz). ^13^C NMR (CDCl_3_, 75 MHz) δ ppm: 29.8 (C9), 56.1 (OMe), 62.3 (C10), 112.1, 116.7, 119.2, 120.2, 125.1, 125.1, 127.4, 129.1, 131.9, 134.2, 135.8, 146.6, 148.2, 150.5 (C2), 159.6, 165.8 (C11). Anal. calcd. for: C_19_H_16_ClNO_3_S: C 61.04, H 4.31, N 3.75; Found: C 61.05, H 4.35, N 3.91. MS: *m*/*z* 374.10. (M+H^+^. 29%).

3-[(7-Chloroquinolin-4-yl)sulfanyl]propyl-4-methoxybenzoate (**23**). Column chromathography DCM:EtOAc (8:2). White solid, yield: 96%; m.p. 106–108 °C; IR (KBr) cm^−1^: 3010, 2926, 1701, 1259; ^1^H NMR (CDCl_3_, 300 MHz) δ ppm: 2.22–2.31 (m, 2H, H10), 3.27 (t, 2H, H9, J = 7.2 Hz), 3.87 (s, 3H, OMe), 4.48 (t, 2H, H11, J = 6.0 Hz), 6.93 (d, 2H, H3′, 5′, J = 9.0 Hz), 7.20 (d, 1H, H3, J = 4.9 Hz), 7.49 (dd, 1H, H6′, J = 2.1, 9.0 Hz), 8.01 (d, 2H, H2′, 6′, J = 9.0 Hz), 8.05 (d, 1H, H8, J = 2.4 Hz), 8.06 (d, 1H, H5, J = 8.8 Hz), 8.68 (d, 1H, H2, J = 4.8 Hz). ^13^C NMR (CDCl_3_, 75 MHz) δ ppm: 28.0 (C10), 28.1 (C9), 55.6 (OMe), 63.0 (C11), 113.9 (C3′,5′), 116.3 (C3), 122.5, 125.2 (C5 or 8), 127.5 (C6), 129.1 (C8 or 5), 131.8 (C2′,6′), 135.9, 147.5, 148.2, 150.4 (C2), 163.7, 166.3 (C12). Anal. calcd. for: C_20_H_18_ClNO_3_S: C 61.93, H 4.68, N 3.61; Found: C 61.96, H 4.71, N 3.72. MS: *m*/*z* 388.10. (M+H^+^. 100%).

3-[(7-Chloroquinolin-4-yl)sulfanyl]propyl-2,3-dimethoxybenzoate (**24**). Column chromathography DCM:EtOAc (8:2). White solid, yield: 91%; m.p. 78–80 °C; IR (KBr) cm^−1^: 3031, 2912, 1728, 1254; ^1^H NMR (CDCl_3_, 300 MHz) δ ppm: 2.24–2.30 (m, 2H, H10), 3.29 (t, 2H, H9, J = 7.3 Hz), 3.89 (s, 3H, OMe), 3.90 (s, 3H, OMe), 4.50 (t, 2H, H11, J = 5.9 Hz), 7.08–7.10 (m, 2H, H5′,4′), 7.21 (d, 1H, H3, J = 4.9 Hz), 7.32 (dd, 1H, H6′, J = 2.7, 6.8 Hz), 7.49 (dd, 1H, H6, J = 2.1, 9.0 Hz), 8.05 (d, 1H, H8, J = 2.5 Hz), 8.06 (d, 1H, H5, J = 8.8 Hz), 8.68 (d, 1H, H2, J = 4.8 Hz). ^13^C NMR (CDCl_3_, 75 MHz) δ ppm: 27.9 (C9,10), 56.2 (OMe), 61.7 (OMe), 63.3 (C11), 116.1 (C3), 116.3 (C4′), 122.3 (C5′), 124.1 (C6′), 125.1 (C5), 125.2, 126.0, 127.4 (C6), 129.1 (C8), 135.8, 147.4, 148.2, 149.2, 150.4 (C2), 153.7, 166.4 (C12). Anal. calcd. for: C_21_H_20_ClNO_4_S: C 60.35, H 4.82, N 3.35; Found: C 60.39, H 4.81, N 3.47. MS: *m*/*z* 418.08. (M+H^+^. 37%).

3-[(7-Chloroquinolin-4-yl)sulfanyl]propyl-2,4-dimethoxybenzoate (**25**). Column chromathography DCM:EtOAc (8:2). White solid, yield: 89%; m.p. 74–76 °C; IR (KBr) cm^−1^: 3040, 2944, 1688, 1280; ^1^H NMR (CDCl_3_, 270 MHz) δ ppm: 2.23–2.30 (m, 2H, H10), 3.33 (t, 2H, H9, J = 7.4 Hz), 3.85 (s, 3H, OMe), 3.86 (s, 3H, OMe), 4.44 (t, 2H, H11, J = 5.9 Hz), 6.48–6.51 (m, 2H, H3′,5′), 7.32 (d, 1H, H3, J = 4.9 Hz), 7.54 (dd, 1H, H6, J = 1.9, 8.9 Hz), 7.84 (d, 1H, H6′, J = 9.2 Hz), 8.08 (d, 1H, H5, J = 8.9 Hz), 8.26 (d, 1H, H8, J = 1.9 Hz), 8.65 (d, 1H, H2, J = 4.9 Hz). ^13^C NMR (CDCl_3_, 67.9 MHz) δ ppm: 27.8 (C10), 28.4 (C9), 55.6 (OMe), 56.1 (OMe), 62.3 (C11), 99.2 (C3′ or 5′), 105.0 (C3′ or 5′), 111.9, 115.0 (C3), 125.1 (C5), 126.5 (C8), 129.2 (C6), 133.9 (C6′), 146.8 (C2), 161.5, 164.7, 165.6 (C12). Anal. calcd. for: C_21_H_20_ClNO_4_S: C 60.35, H 4.82, N 3.35; Found: C 60.33, H 4.84, N 3.51. MS: *m*/*z* 418.11. (M+H^+^. 49%).

3-[(7-Chloroquinolin-4-yl)sulfanyl]propyl-2,5-dimethoxybenzoate (**26**). Column chromathography DCM:EtOAc (8:2). White solid, yield: 96%; m.p. 97–98 °C; IR (KBr) cm^−1^: 2971, 1696, 1286; ^1^H NMR (CDCl_3_, 300 MHz) δ ppm: 2.20–2.29 (m, 2H, H10), 3.29 (t, 2H, H9, J = 7.3 Hz), 3.79 (s, 3H, OMe), 3.84 (s, 3H, OMe), 4.48 (t, 2H, H11, J = 5.9 Hz), 6.93 (d, 1H, H3′, J = 9.1 Hz), 7.05 (dd, 1H, H4′, J = 3.2, 9.1 Hz), 7.23 (d, 1H, H3, J = 4.9 Hz), 7.35 (d, 1H, H6′, J = 3.2 Hz), 7.49 (dd, 1H, H6, J = 2.2, 8.9 Hz), 8.05–8.07 (m, 2H, H5,8), 8.68 (d, 1H, H2, J = 4.9 Hz). ^13^C NMR (CDCl_3_, 75 MHz) δ ppm: 27.9 (C9,10), 56.1 (OMe), 56.8 (OMe), 63.3 (C11), 113.9 (C3′), 116.2 (C3 or 6′), 116.4 (C3 or 6′), 119.6 (C4′), 120.5, 125.1, 125.2 (C5), 127.4 (C8), 129.1 (C6), 135.8, 147.6 (C2), 148.2, 150.4, 153.2, 153.6, 166.2 (C12). Anal. calcd. for: C_21_H_20_ClNO_4_S: C 60.35, H 4.82, N 3.35; Found: C 60.35, H 4.83, N 3.57. MS: *m*/*z* 418.08. (M+H^+^. 61%).

3-[(7-Chloroquinolin-4-yl)sulfanyl]propyl-3,5-dimethoxybenzoate (**27**). White solid, yield: 95% crystallized from ethanol; m.p. 148 °C; IR (KBr) cm^−1^: 2944, 1712, 1248; ^1^H NMR (CDCl_3_, 270 MHz) δ ppm: 2.26–2.31 (m, 2H, H10), 3.30 (t, 2H, H9, J = 7.2 Hz), 3.82 (s, 6H, OMe), 4.49 (t, 2H, H11, J = 5.9 Hz), 6.65 (t, 1H, H4′, J = 2.2 Hz), 7.17 (d, 2H, H2′,6′, J = 2.2 Hz), 7.27 (d, 1H, H3, J = 4.9 Hz), 7.54 (dd, 1H, H6, J = 1.9, 8.6 Hz), 8.08 (d, 1H, H5, J = 8.6 Hz), 8.22 (brs, 1H, H8), 8.68 (d, 1H, H2, J = 4.9 Hz). ^13^C NMR (CDCl_3_, 67.9 MHz) δ ppm: 27.8 (C10), 28.4 (C9), 55.7 (2 × OMe), 63.2 (C11), 105.6 (C4′), 107.5 (C2′,6′), 115.6 (C3), 124.9 (C5), 125.1 (C8), 128.6 (C6), 131.8, 137.8, 147.0 (C2), 152.4, 157.9, 160.9, 166.2 (C12). Anal. calcd. for: C_21_H_20_ClNO_4_S: C 60.35, H 4.82, N 3.35; Found: C 60.39, H 4.79, N 3.60. MS: *m*/*z* 418.12. (M+H^+^. 27%).

3-[(7-Chloroquinolin-4-yl)sulfanyl]propyl-2,3,4-trimethoxybenzoate (**28**). White solid, yield: 85% crystallized from ethanol; m.p. 84 °C; IR (KBr) cm^−1^: 2963, 1692, 1216; ^1^H NMR (CDCl_3_, 270 MHz) δ ppm: 2.20–2.30 (m, 2H, H10), 3.30 (t, 2H, H9, J = 7.2 Hz), 3.87 (s, 3H, OMe), 3.90 (s, 3H, OMe), 3.92 (s, 3H, OMe), 4.46 (t, 2H, H11, J = 5.9 Hz), 6.70 (d, 1H, H5′, J = 8.9 Hz), 7.28 (d, 1H, H3, J = 4.5 Hz), 7.52 (dd, 1H, H6, J = 1.9, 9.2 Hz), 7.59 (d, 1H, H6′, J = 8.9 Hz), 8.07 (d, 1H, H5, J = 9.2 Hz), 8.17 (d, 1H, H8, J = 1.9 Hz), 8.67 (d, 1H, H2, J = 4.5 Hz). ^13^C NMR (CDCl_3_, 67.9 MHz) δ ppm: 27.9 (C10), 28.2 (C9), 56.2 (OMe), 61.0 (OMe), 61.8 (OMe), 62.9 (C11), 107.2 (C5′), 116.0 (C3), 117.7, 125.1 (C5), 126.9 (C6′), 127.7 (C8), 128.0 (C6), 136.9, 143.2, 146.2, 148.6 (C2), 150.1, 154.8, 157.5, 165.5 (C12). Anal. calcd. for: C_22_H_22_ClNO_5_S: C 58.99, H 4.95, N 3.13; Found: C 59.01, H 4.96, N 3.32. MS: *m*/*z* 448.11. (M+H^+^. 100%).

3-[(7-Chloroquinolin-4-yl)sulfanyl]propyl-2,4,5-trimethoxybenzoate (**29**). Column chromathography DCM:EtOAc (8:2). White solid, yield: 58%; m.p. 118–120 °C; IR (KBr) cm^−1^: 3028, 2905, 1714, 1229; ^1^H NMR (CDCl_3_, 300 MHz) δ ppm: 2.18–2.27 (m, 2H, H10), 3.27 (t, 2H, H9, J = 7.3 Hz), 3.84 (s, 3H, OMe), 3.86 (s, 3H, OMe), 3.92 (s, 3H, OMe), 4.45 (t, 2H, H11, J = 6.0 Hz), 6.51 (s, 1H, H3′), 7.20 (d, 1H, H3, J = 4.9 Hz), 7.40 (s, 1H, H6′), 7.46 (dd, 1H, H6, J = 2.3, 8.8 Hz), 8.01 (d, 1H, H8, J = 2.3 Hz), 8.03 (d, 1H, H5, J = 8.8 Hz), 8.64 (d, 1H, H2, J = 4.9 Hz). ^13^C NMR (CDCl_3_, 75 MHz) δ ppm: 27.9 (C9,10), 56.1 (OMe), 56.6 (OMe), 57.0 (OMe), 62.9 (C11), 97.7 (C3′), 110.4, 114.7 (C6′), 116.1 (C3), 125.1 (C5), 125.1, 127.3 (C6), 129.0 (C8), 135.8, 142.7, 147.6, 148.1, 150.3 (C2), 153.9, 155.8, 165.8 (C12). Anal. calcd. for: C_22_H_22_ClNO_5_S: C 58.99, H 4.95, N 3.13; Found: C 58.96, H 4.95, N 3.29. MS: *m*/*z* 448.13. (M+H^+^. 100%).

3-[(7-Chloroquinolin-4-yl)sulfanyl]propyl-3,4,5-trimethoxybenzoate (**30**). Column chromathography DCM:EtOAc (9:1). White solid, yield: 97%; m.p. 98–100 °C; IR (KBr) cm^−1^: 3048, 2925, 1704, 1220; ^1^H NMR (CDCl_3_, 270 MHz) δ ppm: 2.22–2.32 (m, 2H, H10), 3.24 (t, 2H, H9, J = 7.2 Hz), 3.88 (s, 6H, 2 × OMe), 3.89 (s, 3H, OMe), 4.49 (t, 2H, H11, J = 6.2 Hz), 7.17 (d, 1H, H3, J = 4.8 Hz), 7.28 (s, 2H, H2′, 6′), 7.47 (dd, 1H, H6, J = 2.1, 9.0 Hz), 8.03 (d, 1H, H8, J = 2.1 Hz), 8.04 (d, 1H, H5, J = 9.0 Hz), 8.67 (d, 1H, H2, J = 4.8 Hz). ^13^C NMR (CDCl_3_, 67.9 MHz) δ ppm: 27.9 (C10), 28.1 (C9), 56.4 (2 × OMe), 61.1 (OMe), 63.5 (C11), 107.1 (C2′,6′), 116.3 (C3), 125.0 (C5), 125.1, 125.2, 127.5 (C6), 129.1 (C8), 135.9, 142.7, 147.4, 148.2, 150.3 (C2), 153.1, 166.2 (C12). Anal. calcd. for: C_22_H_22_ClNO_5_S: C 58.99, H 4.95, N 3.13; Found: C 58.98, H 4.96, N 3.41. MS: *m*/*z* 448.09. (M+H^+^. 100%).

3-[(7-Chloroquinolin-4-yl)sulfanyl]propyl-2-methoxybenzoate (**31**). Column chromathography DCM:EtOAc (8:2). White solid, yield: 76%; m.p. 95–97 °C; IR (KBr) cm^−1^: 2950, 1714, 1225; ^1^H NMR (CDCl_3_, 270 MHz) δ ppm: 2.25–2.32 (m, 2H, H10), 3.36 (t, 2H, H9, J = 7.2 Hz), 3.88 (s, 3H, OMe), 4.48 (t, 2H, H11, J = 5.9 Hz), 6.97–7.00 (m, 2H, H3′,5′), 7.36 (d, 1H, H3, J = 5.2 Hz), 7.45–7.49 (m, 1H, H4′), 7.57 (dd, 1H, H6, J = 1.9, 8.9 Hz), 7.79 (dd, 1H, H6′, J = 1.7, 7.9 Hz), 8.10 (d, 1H, H5, J = 8.9 Hz), 8.35 (d, 1H, H8, J = 1.9 Hz), 8.65 (d, 1H, H2, J = 5.2 Hz). ^13^C NMR (CDCl_3_, 67.9 MHz) δ ppm: 27.8 (C10), 28.3 (C9), 56.1 (OMe), 62.8 (C11), 112.3 (C3′ or 5′), 115.3 (C3), 120.0 (C3′ or 5′), 120.4, 122.9, 124.8, 125.1 (C5), 125.9 (C8), 128.8 (C6), 131.7 (C6′), 133.9 (C4′), 138.4, 146.5 (C2), 159.3, 166.2 (C12). Anal. calcd. for: C_20_H_18_ClNO_3_S: C 61.93, H 4.68, N 3.61; Found: C 61.95, H 4.68, N 3.69. MS: *m*/*z* 388.10. (M+H^+^. 100%).

3-[(7-Chloroquinolin-4-yl)sulfanyl]propyl-2-chlorobenzoate (**32**). White solid; yield: 81% crystallized from ethanol; m.p. 100–102 °C; IR (KBr) cm^−1^: 2950, 1720, 1230; ^1^H NMR (CDCl_3_, 270 MHz) δ ppm: 2.22–2.31 (m, 2H, H10), 3.29 (t, 2H, H9, J = 7.2 Hz), 4.52 (t, 2H, H11, J = 5.9 Hz), 7.21 (d, 1H, H3, J = 4.9 Hz), 7.29–7.35 (m, 1H, H5′), 7.43–7.46 (m, 2H, H3′,4′), 7.49 (dd, 1H, H6, J = 1.9, 9.2 Hz), 7.80–7.83 (m, 1H, H6′), 8.05 (d, 1H, H5, J = 9.2 Hz), 8.09 (d, 1H, H8, J = 1.9 Hz), 8.68 (brs, 1H, H2). ^13^C NMR (CDCl_3_, 67.9 MHz) δ ppm: 27.8 (C10), 28.2 (C9), 63.8 (C11), 116.3 (C3), 125.1 (C5), 126.7, 127.6, 128.6, 130.2, 131.2, 131.5, 132.8, 133.7, 136.1, 147.4, 148.1, 149.7 (C2), 165.7 (C12). Anal. calcd. for: C_19_H_15_Cl_2_NO_2_S: C 58.17, H 3.85, N 3.57; Found: C 58.16, H 3.87, N 3.73. MS: *m*/*z* 392.02. (M+H^+^. 75%).

3-[(7-Chloroquinolin-4-yl)sulfanyl]propyl-3-chlorobenzoate (**33**). White solid, yield: 80% crystallized from ethanol; m.p. 96–98 °C; IR (KBr) cm^−1^: 2920, 1700, 1210; ^1^H NMR (CDCl_3_, 270 MHz) δ ppm: 2.22–2.32 (m, 2H, H10), 3.26 (t, 2H, H9, J = 7.2 Hz), 4.50 (t, 2H, H11, J = 5.9 Hz), 7.20 (d, 1H, H3, J = 4.9 Hz), 7.38 (t, 1H, H5′, J = 7.9 Hz), 7.49 (dd, 1H, H6, J = 1.9, 9.2 Hz), 7.52–7.56 (m, 1H, H4′), 7.89–7.93 (m, 1H, H6′), 8.00 (t, 1H, H2′, J = 1.7 Hz), 8.05 (d, 1H, H5, J = 9.2 Hz), 8.08 (d, 1H, H8, J = 1.9 Hz), 8.69 (brs, 1H, H2). ^13^C NMR (CDCl_3_, 67.9 MHz) δ ppm: 27.8 (C10), 28.2 (C9), 63.6 (C11), 116.3 (C3), 125.1 (C5), 127.7 (C6), 127.8 (C6′), 128.6 (C8), 129.7 (C2′), 129.8 (C5′), 131.8, 133.3 (C4′), 134.8, 136.2, 143.5, 147.5, 148.1, 149.7 (C2), 165.2 (C12). Anal. calcd. for: C_19_H_15_Cl_2_NO_2_S: C 58.17, H 3.85, N 3.57; Found: C 58.21, H 3.83, N 3.82. MS: *m*/*z* 392.04. (M+H^+^. 63%).

3-[(7-Chloroquinolin-4-yl)sulfanyl]propyl-2,4-dichlorobenzoate (**34**). White solid, yield: 76% crystallized from ethanol; m.p. 88–89 °C; IR (KBr) cm^−1^: 3060, 2930, 1690, 1210; ^1^H NMR (CDCl_3_, 270 MHz) δ ppm: 2.23–2.33 (m, 2H, H10), 3.30 (t, 2H, H9, J = 7.2 Hz), 4.52 (t, 2H, H11, J = 5.9 Hz), 7.25 (d, 1H, H3, J = 4.2 Hz), 7.31 (dd, 1H, H5′, J = 1.9, 8.4 Hz), 7.48 (d, 1H, H3′, J = 1.9 Hz), 7.53 (dd, 1H, H6, J = 1.9, 9.2 Hz), 7.80 (d, 1H, H6′, J = 8.4 Hz), 8.07 (d, 1H, H5, J = 9.2 Hz), 8.19 (d, 1H, H8, J = 1.9 Hz), 8.69 (d, 1H, H2, J = 4.2 Hz). ^13^C NMR (CDCl_3_, 67.9 MHz) δ ppm: 27.6 (C10), 28.2 (C9), 63.9 (C11), 116.0 (C3), 125.1 (C5 or 8),127.2 (C5′), 127.6 (C6 or 3′), 128.1 (C8 or 5), 128.2, 131.2 (C3′ or 6), 132.6 (C6′), 134.8, 137.1, 138.8, 148.3 (C2), 164.8 (C12). Anal. calcd. for: C_19_H_14_Cl_3_NO_2_S: C 53.48, H 3.31, N 3.28; Found: C 53.51, H 3.31, N 3.45. MS: *m*/*z* 426.94. (M+H^+^. 80%).

3-[(7-Chloroquinolin-4-yl)sulfanyl]propyl-4-methoxy-3-nitrobenzoate (**35**). Column chromathography DCM:EtOAc (9:1). White solid, yield: 97%; m.p. 138–140 °C; IR (KBr) cm^−1^: 3020, 2958, 1715, 1507, 1247; ^1^H NMR (CDCl_3_, 300 MHz) δ ppm: 2.24–2.33 (m, 2H, H10), 3.26 (t, 2H, H9, J = 7.1 Hz), 4.03 (s, 3H, OMe), 4.52 (t, 2H, H11, J = 6.1 Hz), 7.13 (d, 1H, H5′, J = 8.8 Hz), 7.19 (d, 1H, H3, J = 4.8 Hz), 7.49 (dd, 1H, H6, J = 2.4, 8.8 Hz), 8.04 (d, 1H, H8, J = 2.4 Hz), 8.06 (d, 1H, H5, J = 8.8 Hz), 8.20 (dd, 1H, H6′, J = 2.2, 8.8 Hz), 8.50 (d, 1H, H2′, J = 2.2 Hz), 8.70 (d, 1H, H2, J = 4.8 Hz). ^13^C NMR (CDCl_3_, 75 MHz) δ ppm: 27.7 (C10), 28.1 (C9), 57.0 (OMe), 63.9 (C11), 113.4 (C5′), 116.4 (C3), 122.4, 125.1 (C5), 125.2, 127.3 (C2′ or 8), 127.5 (C2′ or 8), 129.1 (C6), 135.5 (C6′), 135.9, 139.5, 147.2, 148.2, 150.4 (C2), 156.4, 164.4 (C12). Anal. calcd. for: C_20_H_17_ClN_2_O_5_S: C 55.49, H 3.96, N 6.47; Found: C 55.47, H 4.01, N 6.72. MS: *m*/*z* 433.09. (M+H^+^. 46%).

3-[(7-Chloroquinolin-4-yl)sulfanyl]propyl-5-methyl-2-nitrobenzoate (**36**). Column chromathography DCM:EtOAc (9:1). White solid, yield: 67%; m.p. 91–92 °C; IR (KBr) cm^−1^: 3062, 2971, 1731, 1505, 1205; ^1^H NMR (CDCl_3_, 300 MHz) δ ppm: 2.12–2.21 (m, 2H, H10), 2.39 (s, 3H, CH3), 3.15 (t, 2H, H9, J = 7.2 Hz), 4.46 (t, 2H, H11, J = 5.9 Hz), 7.13 (d, 1H, H3, J = 4.9 Hz), 7.33 (dd, 1H, H4′, J = 1.0, 8.3 Hz), 7.39 (dd, 1H, H6, J = 2.2, 8.94 Hz), 7.44 (d, 1H, H6′, J = 1.0 Hz), 7.79 (d, 1H, H3′, J = 8.3 Hz), 7.94–7.97 (m, 2H, H5,8), 8.62 (d, 1H, H2, J = 4.9 Hz). ^13^C NMR (CDCl_3_, 75 MHz) δ ppm: 21.3 (CH_3_), 27.3 (C10), 27.6 (C9), 64.6 (C11), 116.1 (C3), 124.1 (C3′), 124.9, 125.0 (C5), 127.1 (C6), 127.7, 128.8 (C8), 130.1 (C6′), 132.1 (C4′), 135.5, 144.7, 145.7, 147.2, 147.9, 150.3 (C2), 165.7 (C12). Anal. calcd. for: C_20_H_17_ClN_2_O_4_S: C 57.62, H 4.11, N 6.72; Found: C 57.63, H 4.13, N 6.87. MS: *m*/*z* 417.10. (M+H^+^. 56%).

3-[(7-Chloroquinolin-4-yl)sulfanyl]propyl-3,5-dimethylbenzoate (**37**). Column chromathography DCM:EtOAc (9:1). White solid, yield: 92%; m.p. 136–138 °C; IR (KBr) cm^−1^: 3029, 2772, 1706, 1224; ^1^H NMR (CDCl_3_, 300 MHz) δ ppm: 2.22–2.32 (m, 2H, H10), 2.37 (s, 6H, 2CH_3_), 3.27 (t, 2H, H9, J = 7.2 Hz), 4.49 (t, 2H, H11, J = 6.0 Hz), 7.20–7.26 (m, 2H, H3,4′), 7.49 (dd, 1H, H6, J = 2.1, 9.1 Hz), 7.66 (brs, 2H, H2′,6′), 8.06 (brs, 1H, H8), 8.07 (d, 1H, H5, J = 9.0 Hz), 8.68 (d, 1H, H2, J = 4.8 Hz). ^13^C NMR (CDCl_3_, 75 MHz) δ ppm: 21.3 (2 × CH_3_), 27.9 (C10), 28.0 (C9), 63.2 (C11), 116.3 (C3), 125.1 (C5), 125.2, 127.4 (C2′,5′), 127.5 (C6), 129.1 (C8), 129.9, 135.0 (C3′), 135.9, 138.3, 147.5, 148.2, 150.4 (C2), 166.9 (C11). Anal. calcd. for: C_21_H_20_ClNO_2_S: C 65.36, H 5.22, N 3.63; Found: C 65.39, H 5.24, N 3.79. MS: *m*/*z* 386.11. (M+H^+^. 100%).

3-[(7-Chloroquinolin-4-yl)sulfanyl]propyl-4-(trifluoromethyl)benzoate (**38**). Column chromathography DCM:EtOAc (8:2). White solid, yield: 90%; m.p. 122–124 °C; IR (KBr) cm^−1^: 3049, 2985, 1733, 1236; ^1^H NMR (CDCl_3_, 300 MHz) δ ppm: 2.25–2.34 (m, 2H, H10), 3.27 (t, 2H, H9, J = 7.1 Hz), 4.55 (t, 2H, H11, J = 6.1 Hz), 7.19 (d, 1H, H3, J = 4.8 Hz), 7.49 (dd, 1H, H6, J = 2.3, 9.0 Hz), 7.72 (d, 2H, H3′,5′, J = 8.2 Hz), 8.05 (d, 1H, H8, J = 2.3 Hz), 8.06 (d, 1H, H5, J = 8.9 Hz), 8.16 (d, 2H, H2′,6′, J = 8.2 Hz), 8.70 (d, 1H, H2, J = 4.8 Hz). ^13^C NMR (CDCl_3_, 75 MHz) δ ppm: 27.7 (C10), 28.0 (C9), 63.9 (C11), 116.4 (C3), 125.1 (C5), 125.2, 125.6 (J = 14.8 Hz), 127.5 (C8), 129.1 (C2′ or 6′), 130.1 (C2′ or 6′), 132.2 (J = 4.4 Hz), 133.3, 134.8 (J = 131.4 Hz), 136.0, 147.2, 148.3, 150.4 (C2), 165.4 (C12). Anal. calcd. for: C_20_H_15_ClF_3_NO_2_S: C 56.41, H 3.55, N 3.29; Found: C 56.45, H 3.54, N 3.41. MS: *m*/*z* 426.07. (M+H^+^. 48%).

3-[(7-Chloroquinolin-4-yl)sulfanyl]propyl-4-tert-butylbenzoate (**39**). Column chromathography DCM:EtOAc (8:2). White solid, yield: 80%; m.p. 88–90 °C; IR (KBr) cm^−1^: 3039, 2959, 1702, 1204; ^1^H NMR (CDCl^3^, 270 MHz) δ ppm: 1.34 (s, 9H, 3 × CH_3_), 2.22–2.28 (m, 2H, H10), 3.26 (t, 2H, H9, J = 7.4 Hz), 4.49 (t, 2H, H11, J = 5.9 Hz), 7.20 (d, 1H, H3, J = 4.9 Hz), 7.45–7.50 (m, 3H, H 6,3′,5′), 7.98 (d, 2H, H2′,6′, J = 8.2 Hz), 8.06 (d, 1H, H5, J = 8.9 Hz), 8.06 (d, 1H, H8, J = 1.7 Hz), 8.68 (d, 1H, H2, J = 4.9 Hz). ^13^C NMR (CDCl_3_, 67.9 MHz) δ ppm: 28.0 (C10), 28.2 (C9), 31.2 (3 × CH_3_), 35.2, 63.0 (C11), 116.5 (C3), 125.1 (C6), 125.2, 125.5 (C3′,5′), 127.3, 127.4 (C5), 129.0 (C8), 129.5 (C2′,6′), 135.9, 147.4, 148.2, 150.2 (C2), 157.0, 166.5 (C12). Anal. calcd. for: C_23_H_24_ClNO_2_S: C 66.73, H 5.84, N 3.38; Found: C 66.79, H 5.87, N 3.56. MS: *m*/*z* 414.12. (M+H^+^. 53%).

3-[(7-Chloroquinolin-4-yl)sulfanyl]propyl-2-fluorobenzoate (**40**). White solid, yield: 82% crystallized from ethanol; m.p. 112–113 °C; IR (KBr) cm^−1^: 3084, 2930, 1700, 1225; ^1^H NMR (CDCl_3_, 270 MHz) δ ppm: 2.21–2.31 (m, 2H, H10), 3.30 (t, 2H, H9, J = 7.16 Hz), 4.50 (t, 2H, H11, J = 5.9 Hz), 7.11–7.21 (m, 2H, H3′,5′), 7.24–7.27 (d, 1H, H3, J = 4.2), 7.49 (dd, 1H, H6, J = 1.9, 9.2 Hz), 7.52–7.55 (m, 1H, H4′), 7.94 (td, 1H, H6′, J = 1.7, 7.4 Hz), 8.05 (d, 1H, H5, J = 9.2 Hz), 8.11 (d, 1H, H8, J = 1.9 Hz), 8.67 (d, 1H, H2, J = 4.2 Hz). ^13^C NMR (CDCl_3_, 67.9 MHz) δ ppm: 27.8 (C10), 28.0 (C9), 63.5 (C11), 116.2 (C3), 116.9 (C5′), 117.3, 118.7, 124.2 (C3′ J = 16.5 Hz), 125.1 (C5), 127.8 (C6), 128.2 (C8), 132.3 (C6′), 134.8 (C4′ J = 8.8 Hz), 136.5, 147.0, 149.1, 149.2 (C2), 160.1, 164.5 (C12). Anal. calcd. for: C_19_H1_5_ClFNO_2_S: C 60.72, H 4.02, N 3.73; Found: C 60.73, H 4.02, N 3.90. MS: *m*/*z* 376.05. (M+H^+^. 87%).

#### 3.1.3. General Procedure for the Synthesis of Compounds **41**–**62**

A solution of **3** and the corresponding sulfanyl benzoate derivative **5**, **6**, **8**, **13**, **14**, **17**–**25**, **29**, **30**, **37**, and **38** (0.25 mmol) in dry DCM (5 mL) was treated with m-CPBA (1.2 mmol). The mixture was shaken at room temperature (rt) for 10 min, under a N_2_ atmosphere. The organic layer was washed with an aqueous mixture of saturated sodium bicarbonate and saturated sodium bisulfite (1:1) (3 × 10 mL), water (2 × 20 mL), a saturated NaCl solution (2 × 10 mL), and was finally dried over Na_2_SO_4_, filtered, and evaporated under reduced pressure to give the crude product. The compounds were then purified by column chromatography.

2-[(7-Chloroquinolin-4-yl)sulfinyl]ethanol (**41**). Column chromathography Cyclohexane:Acetone (7:3). White solid, yield: 51%; m.p. 144–146 °C; IR (KBr) cm^−^^1^: 3073, 2982, 1023; ^1^H NMR (DMSO-d_6_, 300 MHz) δ ppm: 2.97–3.04 (m, 1H, H9a), 3.26–3.34 (m, 1H, H9b), 3.75–3.83 (m, 1H, H10a), 3.89–3.99 (m, 1H, H10b), 7.87 (dd, 1H, H6, J = 2.2, 8.9 Hz), 8.03 (d, 1H, H3, J = 4.3 Hz), 8.06 (d, 1H, H5, J = 8.9 Hz), 8.30 (d, 1H, H8, J = 2.2 Hz), 9.21 (d, 1H, H2, J = 4.3 Hz). ^13^C NMR (DMSO-d_6_, 75 MHz) δ ppm: 54.1 (C9), 59.2 (C10), 117.0 (C3), 121.5, 124.2 (C5), 128.5 (C6), 128.6 (C8), 135.0, 147.7, 151.8 (C2), 152.4. Anal. calcd. for: C_11_H_10_ClNO_2_S: C 51.66, H 3.94, N 5.48; Found: C 51.63, H 3.95, N 5.62. MS: *m*/*z* 256.05. (M+H^+^. 78%).

2-[(7-Chloroquinolin-4-yl)sulfinyl]ethyl-4-methoxybenzoate (**42**). Column chromathography DCM:EtOAc (9.5:0.5). Cream solid, yield: 63%; m.p. 126–128 °C; IR (KBr) cm^−1^: 3052, 2929, 1706, 1248, 1020; ^1^H NMR (CDCl_3_, 300 MHz) δ ppm: 3.23–3.31 (m, 1H, H9a), 3.47–3.56 (m, 1H, H9b), 3.84 (s, 3H, OMe), 4.67–4.72 (m, 2H, H10), 6.84 (d, 2H, H3′,5′, J = 8.8 Hz), 7.51 (dd, 1H, H6, J = 2.0, 8.9 Hz), 7.66 (d, 2H, H2′,6′, J = 8.8 Hz), 7.74 (d, 1H, H5, J = 8.9 Hz), 8.01 (d, 1H, H3, J = 4.4 Hz), 8.12 (d, 1H, H8, J = 1.9 Hz), 9.04 (d, 1H, H2, J = 4.4 Hz). ^13^C NMR (CDCl_3_, 75 MHz) δ ppm: 54.9 (C9), 55.6 (OMe), 56.6 (C10), 113.8 (C3′,5′), 117.0 (C3), 121.3, 121.7, 122.6 (C5), 129.1 (C6), 129.8 (C8), 131.6 (C2′,6′), 136.4, 148.4, 150.8, 151.4 (C2), 163.8, 165.6 (C11). Anal. calcd. for: C_19_H_16_ClNO_4_S: C 58.54, H 4.14, N 3.59; Found: C 58.55, H 4.17, N 3.70. MS: *m*/*z* 390.07. (M+H^+^. 71%).

2-[(7-Chloroquinolin-4-yl)sulfinyl]ethyl-2,3-dimethoxybenzoate (**43**). Column chromathography DCM:EtOAc (5:5). Cream solid, yield: 93%; m.p. 110 °C; IR (KBr) cm^−1^: 3087, 2939, 1721, 1228, 1031; ^1^H NMR (CDCl_3_, 300 MHz) δ ppm: 3.16–3.23 (m, 1H, H9a), 3.46–3.55 (m, 1H, H9b), 3.86 (s, 3H, OMe), 3.87 (s, 3H, OMe), 4.70–4.75 (m, 2H, H10), 7.03–7.10 (m, 3H, H4′,5′,6′), 7.52 (dd, 1H, H6, J = 2.1, 8.9 Hz), 7.78 (d, 1H, H5, J = 8.9 Hz), 8.01 (d, 1H, H3, J = 4.4 Hz), 8.13 (d, 1H, H8, J = 2.1 Hz), 9.05 (d, 1H, H2, J = 4.4 Hz). ^13^C NMR (CDCl_3_, 75 MHz) δ ppm: 55.5 (C9), 56.1 (OMe), 57.4 (C10), 61.6 (OMe), 116.4, 116.8 (C3), 121.7, 122.1 (C5′), 122.7 (C5), 124.0 (C6′), 124.9, 129.1 (C6), 129.7 (C8), 136.4, 148.4, 149.3, 151.0, 151.4 (C2), 153.6, 165.5 (C11). Anal. calcd. for: C_20_H_18_ClNO_5_S: C 57.21, H 4.32, N 3.34; Found: C 57.16, H 4.32, N 3.59. MS: *m*/*z* 420.07. (M+H^+^. 61%).

2-[(7-Chloroquinolin-4-yl)sulfinyl]ethyl-2,5-dimethoxybenzoate (**44**). Column chromathography DCM:EtOAc (8:2). Cream solid, yield: 85%; m.p. 112–113 °C; IR (KBr) cm^−1^: 3056, 2960, 1724, 1204, 1038; ^1^H NMR (CDCl_3_, 300 MHz) δ ppm: 3.20–3.28 (m, 1H, H9a), 3.44–3.53 (m, 1H, H9b), 3.75 (s, 3H, OMe), 3.79 (s, 3H, OMe), 4.67–4.70 (m, 2H, H10), 6.86 (d, 1H, H3′, J = 9.0 Hz), 7.02 (dd, 1H, H4′, J = 3.2, 9.0 Hz), 7.09 (d, 1H, H6′, J = 3.2 Hz), 7.49 (dd, 1H, H6, J = 2.1, 8.9 Hz), 7.75 (d, 1H, H5, J = 8.9 Hz), 8.00 (d, 1H, H3, J = 4.4 Hz), 8.08 (d, 1H, H8, J = 2.1 Hz), 9.02 (d, 1H, H2, J = 4.4 Hz). ^13^C NMR (CDCl_3_, 75 MHz) δ ppm: 55.3 (C9), 55.9 (OMe), 56.6 (OMe), 57.1 (C10), 113.7 (C3′), 116.2, 116.8 (C3), 119.0, 120.0 (C4′), 121.7, 122.6 (C5), 129.0 (C6), 129.7 (C8), 136.3, 148.3, 150.9, 151.3 (C2), 152.9, 153.7, 165.2 (C11). Anal. calcd. for: C_20_H_18_ClNO_5_S: C 57.21, H 4.32, N 3.34; Found: C 57.22, H 4.31, N 3.43. MS: *m*/*z* 420.05. (M+H^+^. 77%).

2-[(7-Chloroquinolin-4-yl)sulfinyl]ethyl-2,4,5-trimethoxybenzoate (**45**). Column chromathography DCM:EtOAc (8:2). White solid, yield: 91%; m.p. 151–153 °C; IR (KBr) cm^−1^: 3071, 2940, 1711, 1204, 1022; ^1^H NMR (CDCl_3_, 300 MHz) δ ppm: 3.22–3.30 (m, 1H, H9a), 3.44–3.53 (m, 1H, H9b), 3.82 (s, 3H, OMe), 3.85 (s, 3H, OMe), 3.94 (s, 3H, OMe), 4.67 (t, 2H, H10, J = 4.9 Hz), 6.48 (s, 1H, H3′), 7.20 (s, 1H, H6′), 7.48 (dd, 1H, H6, J = 2.1, 8.9 Hz), 7.76 (d, 1H, H5, J = 8.9 Hz), 8.02 (d, 1H, H3, J = 4.4 Hz), 8.10 (d, 1H, H8, J = 2.1 Hz), 9.04 (d, 1H, H2, J = 4.4 Hz). ^13^C NMR (CDCl_3_, 75 MHz) δ ppm: 55.5 (C9), 56.2 (OMe), 56.5 (OMe), 56.8 (OMe), 56.9 (C10), 97.4 (C3′), 109.2, 114.4 (C6′), 116.9 (C3), 121.7, 122.7 (C5), 129.0 (C6), 129.7 (C8), 136.3, 142.6, 148.4, 151.0, 151.4 (C2), 154.3, 156.1, 164.9 (C12). Anal. calcd. for: C_21_H_20_ClNO_6_S: C 56.06, H 4.48, N 3.11; Found: C 56.04, H 4.50, N 3.27. MS: *m*/*z* 450.11. (M+H^+^. 83%).

2-[(7-Chloroquinolin-4-yl)sulfinyl]ethyl-3,4,5-trimethoxybenzoate (**46**). Column chromathography DCM:EtOAc (8:2). Pink solid, yield: 83%; m.p. 144–146 °C; IR (KBr) cm^−^^1^: 3076, 2960, 1706, 1222, 1044; ^1^H NMR (CDCl_3_, 300 MHz) δ ppm: 3.24–3.32 (m, 1H, H9a), 3.45–3.54 (m, 1H, H9b), 3.87 (s, 6H, 2 × OMe), 3.87 (s, 3H, OMe), 4.66–4.71 (m, 2H, H10), 6.99 (s, 2H, H2′,6′), 7.46 (dd, 1H, H6, J = 2.1, 8.9 Hz), 7.68 (d, 1H, H5, J = 8.9 Hz), 7.98 (d, 1H, H3, J = 4.4 Hz), 8.05 (d, 1H, H8, J = 2.1 Hz), 9.01 (d, 1H, H2, J = 4.4 Hz). ^13^C NMR (CDCl_3_, 75 MHz) δ ppm: 54.6 (C9), 56.2 (2 × OMe), 57.1 (C10), 60.9 (OMe), 106.8 (C2′,6′), 116.9 (C3), 121.5, 122.4 (C5), 123.8, 128.9 (C6), 129.7 (C8), 136.3, 142.7, 148.3, 150.6, 151.3 (C2), 152.9, 165.5 (C11). Anal. calcd. for: C_21_H_20_ClNO_6_S: C 56.06, H 4.48, N 3.11; Found: C 56.05, H 4.47, N 3.23. MS: *m*/*z* 450.14. (M+H^+^. 100%).

2-[(7-Chloroquinolin-4-yl)sulfinyl]ethyl-2-methoxybenzoate (**47**). Column chromathography DCM:EtOAc (9:1). Pink solid, yield: 78%; m.p. 105–107 °C; IR (KBr) cm^−1^: 3079, 2965, 1719, 1240, 1043; ^1^H NMR (CDCl_3_, 300 MHz) δ ppm: 3.22–3.29 (m, 1H, H9a), 3.46–3.55 (m, 1H, H9b), 3.85 (s, 3H, OMe), 4.68–4.70 (m, 2H, H10), 6.89–6.96 (m, 2H, H3′,5′), 7.45–7.51 (m, 3H, H4′,6′,6), 7.76 (d, 1H, H5, J = 8.9 Hz), 8.02 (d, 1H, H3, J = 4.2 Hz), 8.11 (d, 1H, H8, J = 2.0 Hz), 9.03 (d, 1H, H2, J = 4.2 Hz). ^13^C NMR (CDCl_3_, 75 MHz) δ ppm: 55.2 (C9), 56.0 (OMe), 56.9 (C10), 112.1, 116.9 (C3), 118.6, 120.2, 121.7, 122.7 (C5), 129.0 (C6), 129.7 (C8), 131.7, 134.3, 136.3, 148.4, 151.0, 151.4 (C2), 159.4, 165.3 (C12). Anal. calcd. for: C_19_H_16_ClNO_4_S: C 58.53, H 4.14, N 3.59; Found: C 58.55, H 4.17, N 3.72. MS: *m*/*z* 390.08. (M+H^+^. 100%).

2-[(7-Chloroquinolin-4-yl)sulfinyl]ethyl-4-methoxy-3-nitrobenzoate (**48**). Column chromathography DCM:EtOAc (9:1). Pink solid, yield: 88%; m.p. 148–150 °C; IR (KBr) cm^−1^: 3035, 2981, 1721, 1527, 1238, 1052; ^1^H NMR (CDCl_3_, 300 MHz) δ ppm: 3.27–3.35 (m, 1H, H9a), 3.49–3.57 (m, 1H, H9b), 4.01 (s, 3H, OMe), 4.70–4.75 (m, 2H, H10), 7.06 (d, 1H, H5′, J = 8.9 Hz), 7.56 (dd, 1H, H6, J = 2.1, 8.9 Hz), 7.75 (d, 1H, H5, J = 8.9 Hz), 7.85 (dd, 1H, H6′, J = 2.2, 8.9 Hz), 7.99 (d, 1H, H3, J = 4.4 Hz), 8.08 (d, 1H, H8, J = 2.1 Hz), 8.13 (d, 1H, H2′, J = 2.2 Hz), 9.02 (d, 1H, H2, J = 4.4 Hz). ^13^C NMR (CDCl_3_, 75 MHz) δ ppm: 54.1 (C9), 57.0 (OMe), 57.3 (C10), 113.3 (C5′), 117.0 (C3), 121.2, 121.5, 122.5 (C5), 127.1 (C2′), 129.2 (C6), 129.7 (C8), 135.2 (C6′), 136.5, 139.1, 148.3, 150.5, 151.4 (C2), 156.5, 163.7 (C12). Anal. calcd. for: C_19_H_15_ClN_2_O_6_S: C 52.48, H 3.48, N 6.44; Found: C 52.49, H 3.48, N 6.62. MS: *m*/*z* 435.07. (M+H^+^. 80%).

2-[(7-Chloroquinolin-4-yl)sulfinyl]ethyl-5-methyl-2-nitrobenzoate (**49**). Column chromathography DCM:EtOAc (9:1). Pink solid, yield: 92%; m.p. 81–83 °C; IR (KBr) cm^−1^: 3067, 2969, 1731, 1526, 1200, 1065; ^1^H NMR (CDCl_3_, 300 MHz) δ ppm: 2.45 (s, 3H, CH_3_), 3.07–3.14 (m, 1H, H9a), 3.43–3.53 (m, 1H, H9b), 4.74–4.79 (m, 2H, H10), 7.30 (brs, 1H, H6′), 7.39–7.42 (m, 1H, H4′), 7.58 (dd, 1H, H6, J = 2.1, 8.9 Hz), 7.82 (d, 1H, H3′, J = 8.3 Hz), 7.85 (d, 1H, H5, J = 8.9 Hz), 7.98 (d, 1H, H3, J = 4.4 Hz), 8.09 (d, 1H, H8, J = 2.1 Hz), 9.03 (d, 1H, H2, J = 4.4 Hz). ^13^C NMR (CDCl_3_, 75 MHz) δ ppm: 21.4 (CH_3_), 54.5 (C9), 58.1 (C10), 116.7 (C3), 121.6, 123.0 (C5), 124.2, 127.1, 129.2 (C6), 129.5 (C8), 130.1, 132.3, 136.5, 145.0, 148.3, 151.1, 151.3 (C2), 165.2 (C11). Anal. calcd. for: C_19_H_15_ClN_2_O_5_S: C 54.48, H 3.61, N 6.69; Found: C 54.45, H 3.59, N 6.83. MS: *m*/*z* 419.10. (M+H^+^. 100%).

2-[(7-Chloroquinolin-4-yl)sulfinyl]ethyl-3,5-dimethylbenzoate (**50**). Column chromathography DCM:EtOAc (8:2). White solid, yield: 83%; m.p. 106–108 °C; IR (KBr) cm^−1^: 3032, 2971, 1707, 1221, 1041; ^1^H NMR (CDCl_3_, 300 MHz) δ ppm: 2.32 (s, 6H, CH_3_), 3.28–3.36 (m, 1H, H9a), 3.50–3.59 (m, 1H, H9b), 4.66–4.80 (m, 2H, H10), 7.17 (brs, 1H, H4′), 7.30 (brs, 2H, H2′,6′), 7.52 (dd, 1H, H6, J = 2.0, 8.9 Hz), 7.75 (d, 1H, H5, J = 8.9 Hz), 8.03 (d, 1H, H3, J = 4.4 Hz), 8.11 (d, 1H, H8, J = 1.9 Hz), 9.05 (d, 1H, H2, J = 4.4 Hz). ^13^C NMR (CDCl_3_, 75 MHz) δ ppm: 21.2 (2 × CH_3_), 54.6 (C9), 56.6 (C10), 117.1 (C3), 121.7, 122.6 (C5), 127.2 (C2′,6′), 128.8 (C6), 129.1, 129.8 (C8), 135.3 (C4′), 136.4, 138.2, 148.4, 150.8, 151.3 (C2), 166.2 (C11). Anal. calcd. for: C_20_H_18_ClNO_3_S: C 61.93, H 4.68, N 3.61; Found: C 61.93, H 4.69, N 3.77. MS: *m*/*z* 388.12. (M+H^+^. 100%).

2-[(7-Chloroquinolin-4-yl)sulfinyl]ethyl-4-(trifluoromethyl)benzoate (**51**). Column chromathography DCM:EtOAc (8:2). White solid, yield: 78%; m.p. 148–150 °C; IR (KBr) cm^−1^: 3070, 2970, 1727, 1220, 1036; ^1^H NMR (CDCl_3_, 270 MHz) δ ppm: 3.27–3.36 (m, 1H, H9a), 3.50–3.60 (m, 1H, H9b), 4.78 (t, 2H, H10, J = 4.9 Hz), 7,56 (dd, 1H, H6, J = 1.9, 8.9 Hz), 7,66 (d, 2H, H, 3′,5′ J = 8.7 Hz), 7.74 (d, 1H, H5, J = 8,9 Hz), 7.84 (d, 2H, H2′,6′, J = 8.7 Hz), 8.05 (d, 1H, H3, J = 4.4 Hz), 8.13 (d, 1H, H8, J = 1.9 Hz), 9.08 (d, 1H, H2, J = 4.4 Hz). ^13^C NMR (CDCl_3_, 67.9 MHz) δ ppm: 54.4 (C9), 57.5 (C10), 117.1 (C3), 121.7, 122.4 (C5), 125.5 (C3′ or 5′), 125.6 (C3′ or 5′), 129.2 (C6), 129.9 (C8,2′,6′), 132.3, 134.8, 135.3, 136.6, 148.5, 150.7, 151.3 (C2), 164.7 (C11). Anal. calcd. for: C_19_H_13_ClF_3_NO_3_S: C 53.34, H 3.06, N 3.27; Found: C 53.37, H 3.08, N 3.45. MS: *m*/*z* 428.11. (M+H^+^. 100%).

2-[(7-Chloroquinolin-4-yl)sulfinyl]ethyl-4-tert-butylbenzoate (**52**). Column chromathography DCM:EtOAc (7:3). White solid, yield: 82%; m.p. 106 °C; IR (KBr) cm^−1^: 3081, 2975, 1726, 1267, 1015; ^1^H NMR (CDCl^3^, 300 MHz) δ ppm: 1.35 (s, 9H, CH3), 3.26–3.34 (m, 1H, H9a), 3.49–3.58 (m, 1H, H9b), 4.73 (t, 2H, H10, J = 5.0 Hz), 7.41 (d, 2H, H3′,5′, J = 8.4 Hz), 7.53 (dd, 1H, H6, J = 2.1, 8.9 Hz), 7.66 (d, 2H, H2′,6′, J = 8.4 Hz), 7.75 (d, 1H, H5, J = 8.9 Hz), 8.05 (d, 1H, H3, J = 4.4 Hz), 8.12 (d, 1H, H8, J = 2.1 Hz), 9.08 (d, 1H, H2, J = 4.4 Hz). ^13^C NMR (CDCl_3_, 75 MHz) δ ppm: 31.2 (CH3), 35.3 (C12), 55.1 (C9), 56.8 (C10), 117.1 (C3), 121.8, 122.6 (C5), 125.6 (C3′,5′), 126.2, 129.1 (C6 or 8), 129.5 (C2′,6′), 129.9 (C6 or 8), 136.5, 148.5, 150.8, 151.5 (C2), 157.4, 166.0 (C11). Anal. calcd. for: C_22_H_22_ClNO_3_S: C 63.53, H 5.33, N 3.37; Found: C 63.52, H 5.34, N 3.49. MS: *m*/*z* 416.15. (M+H^+^. 100%).

3-[(7-Chloroquinolin-4-yl)sulfinyl]propyl-4-methoxybenzoate (**53**). Column chromathography DCM:EtOAc (5:5). Cream solid, yield: 63%; m.p. 126–128 °C; IR (KBr) cm^−1^: 3078, 2962, 1717, 1257, 1016; ^1^H NMR (CDCl_3_, 300 MHz) δ ppm: 1.97–2.10 (m, 1H, H10a), 2.30–2.48 (m, 1H, H10b), 2.88–3.04 (m, 1H, H9a), 3.19–3.29 (m, 1H, H9b), 3.83 (s, 3H, OMe), 4.31–4.47 (m, 2H, H11), 6.86 (d, 2H, H3′,5′, J = 8.9 Hz), 7.46 (dd, 1H, H6, J = 2.1, 8.9 Hz), 7.74 (d, 1H, H5, J = 8.9 Hz), 7.83 (d, 2H, H2′,6′, J = 8.9 Hz), 7.98 (d, 1H, H3, J = 4.4 Hz), 8.17 (d, 1H, H8, J = 2.1 Hz), 9.08 (d, 1H, H2, J = 4.4 Hz). ^13^C NMR (CDCl_3_, 75 MHz) δ ppm: 21.8 (C10), 52.2 (C9), 55.5 (OMe), 62.5 (C11), 113.8 (C3′,5′), 117.1 (C3), 121.7, 122.0, 122.8 (C5), 128.9 (C6), 129.8 (C8), 131.6 (C2′,6′), 136.4, 148.5, 150.9, 151.3 (C2), 163.7, 166.0 (C12). Anal. calcd. for: C_20_H_18_ClNO_4_S: C 59.48, H 4.49, N 3.47; Found: C 59.45, H 4.49, N 3.53. MS: *m*/*z* 404.14. (M+H^+^. 100%).

3-[(7-Chloroquinolin-4-yl)sulfinyl]propyl-2,3-dimethoxybenzoate (**54**). Column chromathography DCM:EtOAc (9:1). Cream solid, yield: 94%; m.p. 111–112 °C; IR (KBr) cm^−1^: 3070, 2922, 1701, 1259, 1051; ^1^H NMR (CDCl_3_, 300 MHz) δ ppm: 2.00–2.14 (m, 1H, H10a), 2.33–2.47 (m, 1H, H10b), 2.90–3.00 (m, 1H, H9a), 3.24–3.34 (m, 1H, H9b), 3.77 (s, 3H, OMe), 3.88 (s, 3H, OMe), 4.37–4.54 (m, 2H, H11), 7.05 (d, 2H, H4′,5′, J = 4.8 Hz), 7.19 (t, 1H, H6′, J = 4.9 Hz), 7.49 (dd, 1H, H6, J = 1.4, 8.9 Hz), 7.77 (d, 1H, H5, J = 8.9 Hz), 8.00 (d, 1H, H3, J = 4.4 Hz), 8.20 (brs 1H, H8), 9.10 (d, 1H, H2, J = 4.4 Hz). ^13^C NMR (CDCl_3_, 75 MHz) δ ppm: 21.9 (C10), 52.2 (C9), 56.2 (OMe), 61.5 (OMe), 62.9 (C11), 116.1, 117.1 (C3), 121.8, 122.1 (C5), 123.0, 124.0, 125.7, 129.1 (C6), 129.8 (C8), 136.5, 148.6, 149.1, 151.0, 151.3 (C2), 153.7, 166.2 (C12). Anal. calcd. for: C_21_H_20_ClNO_5_S: C 58.13, H 4.65, N 3.23; Found: C 58.10, H 4.67, N 3.29. MS: *m*/*z* 434.12. (M+H^+^. 100%).

3-[(7-Chloroquinolin-4-yl)sulfinyl]propyl-,5-dimethoxybenzoate (**55**). Column chromathography DCM:EtOAc (9:1). Clear oil, yield: 73%; IR (NaCl) cm^−1^: 3054, 2947, 1699, 1217, 1047; ^1^H NMR (CDCl_3_, 300 MHz) δ ppm: 2.00–2.14 (m, 1H, H10a), 2.31–2.45 (m, 1H, H10b), 2.89–2.98 (m, 1H, H9a), 3.23–3.32 (m, 1H, H9b), 3.68 (s, 3H, OMe), 3.75 (s, 3H, OMe), 4.35–4.52 (m, 2H, H11), 6.86 (d, 1H, H3′, J = 9.1 Hz), 7.00 (dd, 1H, H4′, J = 3.2, 9.1 Hz), 7.22 (d, 1H, H6′, J = 3.2 Hz), 7.47 (dd, 1H, H6, J = 2.1, 8.9 Hz), 7.77 (d, 1H, H5, J = 8.9 Hz), 7.99 (d, 1H, H3, J = 4.4 Hz), 8.18 (d, 1H, H8, J = 2.1 Hz), 9.09 (d, 1H, H2, J = 4.4 Hz). ^13^C NMR (CDCl_3_, 75 MHz) δ ppm: 22.0 (C10), 52.5 (C9), 55.9 (OMe), 56.5 (OMe), 62.7 (C11), 113.7 (C3′), 116.4, 117.0 (C3), 119.6, 120.0, 121.7, 122.9 (C5), 129.0 (C6), 129.7 (C8), 136.4, 148.5, 151.1, 151.3 (C2), 153.1, 153.5, 165.8 (C12). Anal. calcd. for: C_21_H_20_ClNO_5_S: C 58.13, H 4.65, N 3.23; Found: C 58.17, H 4.69, N 3.42. MS: *m*/*z* 434.10. (M+H^+^. 89%).

3-[(7-Chloroquinolin-4-yl)sulfinyl]propyl-2,4,5-trimethoxybenzoate (**56**). Column chromathography DCM:EtOAc (8:2). Yellow oil, yield: 79%; IR (NaCl) cm^−1^: 3071, 2940, 1711, 1204, 1022; ^1^H NMR (CDCl_3_, 300 MHz) δ ppm: 2.01–2.08 (m, 1H, H10a), 2.33–2.42 (m, 1H, H10b), 2.89–2.98 (m, 1H, H9a), 3.22–3.30 (m, 1H, H9b), 3.70 (s, 3H, OMe), 3.80 (s, 3H, OMe), 3.91 (s, 3H, OMe), 4.35–4.56 (m, 2H, H11), 6.45 (s, 1H, H3′), 7.31 (s, 1H, H6′), 7.45 (dd, 1H, H6, J = 1.5, 8.9 Hz), 7.76 (d, 1H, H5, J = 8.9 Hz), 8.00 (d, 1H, H3, J = 4.2 Hz), 8.18 (d, 1H, H8, J = 1.5 Hz), 9.09 (d, 1H, H2, J = 4.2 Hz). ^13^C NMR (CDCl_3_, 75 MHz) δ ppm: 22.1 (C10), 52.8 (C9), 56.1 (OMe), 56.7 (OMe), 56.9 (OMe), 62.4 (C11), 98.0 (C3′), 110.4, 115.0 (C6′), 117.0 (C3), 121.8, 122.9 (C5), 128.9 (C6), 129.8 (C8), 136.4, 142.9, 148.6, 151.3 (C2), 151.4, 154.2, 155.8, 165.5 (C12). Anal. calcd. for: C_22_H_22_ClNO_6_S: C 56.96, H 4.78, N 3.02; Found: C 57.01, H 4.78, N 2.97. MS: *m*/*z* 464.13. (M+H^+^. 100%).

3-[(7-Chloroquinolin-4-yl)sulfinyl]propyl-3,4,5-trimethoxybenzoate (**57**). Column chromathography DCM:EtOAc (9:1). Cream solid, yield: 75%; m.p. 148 °C; IR (KBr) cm^−1^: 3016, 2993, 1613, 1238, 1021; ^1^H NMR (CDCl_3_, 300 MHz) δ ppm: 1.98–2.11 (m, 1H, H10a), 2.33–2.46 (m, 1H, H10b), 2.86–2.95 (m, 1H, H9a), 3.16–3.26 (m, 1H, H9b), 3.82 (s, 3H, OMe), 3.86 (s, 6H, OMe), 4.32–4.50 (m, 2H, H11), 7.17 (brs, 2H, H2′,6′), 7.47 (dd, 1H, H6, J = 1.9, 8.9 Hz), 7.74 (d, 1H, H5, J = 8.9 Hz), 7.97 (d, 1H, H3, J = 4.4 Hz), 8.15 (d, 1H, H8, J = 1.9 Hz), 9.07 (d, 1H, H2, J = 4.4 Hz). ^13^C NMR (CDCl_3_, 75 MHz) δ ppm: 21.8 (C10), 52.2 (C9), 56.3 (OMe), 61.0 (2 × OMe), 63.1 (C11), 106.9 (C2′,6′), 117.0 (C3), 121.6, 122.7 (C5), 124.5, 128.9 (C6), 129.8 (C8), 136.4, 142.6, 148.5, 150.9, 151.3 (C2), 153.0, 165.9 (C12). Anal. calcd. for: C_22_H_22_ClNO_6_S: C 56.96, H 4.78, N 3.02; Found: C 56.93, H 4.77, N 3.19. MS: *m*/*z* 464.09. (M+H^+^. 100%).

3-[(7-Chloroquinolin-4-yl)sulfinyl]propyl-2-methoxybenzoate (**58**). Column chromathography DCM:EtOAc (8:2). Brown oil, yield: 78%; IR (NaCl) cm^−1^: 3044, 2963, 1735, 1237, 1056; ^1^H NMR (CDCl_3_, 300 MHz) δ ppm: 1.97–2.12 (m, 1H, H10a), 2.40–2.47 (m, 1H, H10b), 2.88–3.05 (m, 1H, H9a), 3.21–3.31 (m, 1H, H9b), 3.72 (s, 3H, OMe), 4.32–4.49 (m, 2H, H11), 6.89–6.94 (m, 2H, H3′ or 4′ or 5′ or 6′), 7.41–7.47 (m, 2H, H3′ or 4′ or 5′ or 6′), 7.65 (dd, 1H, H6, J = 1.9, 8.9 Hz), 7.75 (d, 1H, H5, J = 8.9 Hz), 7.98 (d, 1H, H3, J = 4.4 Hz), 8.17 (d, 1H, H8, J = 1.9 Hz), 9.07 (d, 1H, H2, J = 4.4 Hz). ^13^C NMR (CDCl_3_, 75 MHz) δ ppm: 21.9 (C10), 52.5 (C9), 55.8 (OMe), 62.5 (C11), 112.1 (C3′ or 5′), 117.0 (C3), 119.5, 120.2 (C3′ or 5′), 121.7, 122.9 (C5), 128.9 (C6), 129.8 (C8), 131.7 (C6′), 133.9 (C4′), 136.4, 148.5, 151.1, 151.3 (C2), 159.2, 166.0 (C12). Anal. calcd. for: C_20_H_18_ClNO_4_S: C 59.48, H 4.49, N 3.47; Found: C 59.52, H 4.50, N 3.58. MS: *m*/*z* 404.13. (M+H^+^. 100%).

3-[(7-Chloroquinolin-4-yl)sulfinyl]propyl-4-methoxy-3-nitrobenzoate (**59**). Column chromathography DCM:EtOAc (9:1). Cream solid, yield: 80%; m.p. 124 °C; IR (KBr) cm^−1^: 3077, 2948, 1707, 1532, 1232, 1072; ^1^H NMR (CDCl_3_, 300 MHz) δ ppm: 1.97–2.11 (m, 1H, H10a), 2.30–2.44 (m, 1H, H10b), 2.88–2.98 (m, 1H, H9a), 3.20–3.29 (m, 1H, H9b), 3.99 (s, 3H, OMe), 4.34–4.48 (m, 2H, H11), 7.08 (d, 1H, H5′, J = 8.9 Hz), 7.52 (dd, 1H, H6, J = 2.1, 8.9 Hz), 7.76 (d, 1H, H5, J = 8.9 Hz), 7.98 (d, 1H, H3, J = 4.4 Hz), 8.03 (dd, 1H, H6′, J = 2.2, 8.8 Hz), 8.14 (d, 1H, H8, J = 2.0 Hz), 8.32 (d, 1H, H2′, J = 2.1 Hz), 9.08 (d, 1H, H2, J = 4.4 Hz). ^13^C NMR (CDCl^3^, 75 MHz) δ ppm: 21.6 (C10), 51.6 (C9), 57.0 (OMe), 63.4 (C11), 113.3 (C5′), 117.2 (C3), 121.6, 121.9, 122.7 (C5), 127.2 (C2′), 129.0 (C6), 129.8 (C8), 135.3 (C6′), 136.4, 139.3, 148.5, 150.7, 151.3 (C2), 156.3, 164.1 (C12). Anal. calcd. for: C_20_H_17_ClN_2_O_6_S: C 53.51, H 3.82, N 6.24; Found: C 53.51, H 3.79, N 6.37. MS: *m*/*z* 449.11. (M+H^+^. 98%).

3-[(7-Chloroquinolin-4-yl)sulfinyl]propyl-5-methyl-2-nitrobenzoate (**60**). Column chromathography DCM:EtOAc (9:1). Cream solid, yield: 69%; m.p. 116 °C; IR (KBr) cm^−1^: 3039, 2987, 1736, 1521, 1200, 1063; ^1^H NMR (CDCl_3_, 300 MHz) δ ppm: 1.95–2.08 (m, 1H, H10a), 2.29–2.40 (m, 1H, H10b), 2.42 (s, 3H, CH3), 2.83–2.92 (m, 1H, H9a), 3.17–3.27 (m, 1H, H9b), 4.31–4.39 (m, 1H, H11a), 4.47–4.54 (m, 1H, H11b), 7.36–7.38 (m, 2H, H4′,5), 7.54 (dd, 1H, H6, J = 2.1, 8.9 Hz), 7.81 (d, 2H, H3′,6′, J = 8.9 Hz), 7.97 (d, 1H, H3, J = 4.1 Hz), 8.15 (d, 1H, H8, J = 2.1 Hz), 9.07 (d, 1H, H2, J = 4.4 Hz). ^13^C NMR (CDCl_3_, 75 MHz) δ ppm: 21.4 (CH3), 21.6 (C10), 51.8 (C9), 64.4 (C11), 117.0 (C3), 121.7, 123.1 (C5), 124.1 (C3′), 127.7, 129.0 (C6), 129.6 (C8), 130.1 (C6′), 132.2 (C4′), 136.4, 144.9, 145.4, 148.5, 150.9, 151.2 (C2), 165.7 (C12). Anal. calcd. for: C_20_H_17_ClN_2_O_5_S: C 55.49, H 3.96, N 6.47; Found: C 55.47, H 3.97, N 6.61. MS: *m*/*z* 433.10. (M+H^+^. 96%).

3-[(7-Chloroquinolin-4-yl)sulfinyl]propyl-3,5-dimethylbenzoate (**61**). Column chromathography DCM:EtOAc (1:1). Cream solid, yield: 75%; m.p. 105–107 °C; IR (KBr) cm^−1^: 3070, 2989, 1715, 1218, 1037; ^1^H NMR (CDCl_3_, 300 MHz) δ ppm: 2.00–2.14 (m, 1H, H10a), 2.34–2.46 (m, 1H, H10b), 2.31 (s, 6H, CH_3_), 2.89–2.98 (m, 1H, H9a), 3.21–3.31 (m, 1H, H9b), 4.34–4.42 (m, 1H, H11a), 4.43–4.51 (m, 1H, H11b), 7.17 (brs, 1H, H4′), 7.45 (dd, 1H, H6, J = 2.1, 8.9 Hz), 7.52 (brs, 2H, H2′,6′), 7.74 (d, 1H, H5, J = 8.9 Hz), 7.99 (d, 1H, H3, J = 4.4 Hz), 8.17 (d, 1H, H8, J = 2.1 Hz), 9.09 (d, 1H, H2, J = 4.4 Hz). ^13^C NMR (CDCl_3_, 75 MHz) δ ppm: 21.2 (2 × CH_3_), 21.9 (C10), 52.3 (C9), 62.6 (C11), 117.1 (C3), 121.7, 121.8, 122.8 (C5), 127.3 (C2′,6′), 128.9 (C6), 129.5, 129.8 (C8), 135.0 (C4′), 136.4, 138.2, 148.5, 150.9, 151.4 (C2), 166.6 (C12). Anal. calcd. for: C_21_H_20_ClNO_3_S: C 62.76, H 5.02, N 3.49; Found: C 62.77, H 4.98, N 3.65. MS: *m*/*z* 402.15. (M+H^+^. 100%).

3-[(7-Chloroquinolin-4-yl)sulfinyl]propyl-4-(trifluoromethyl)benzoate (**62**). Column chromathographyc DCM:EtOAc (8:2). White solid, yield: 68%; m.p. 86–87 °C; IR (KBr) cm^−1^: 2958, 1730, 1280, 1020; ^1^H NMR (CDCl_3_, 300 MHz) δ ppm: 1.99–2.13 (m, 1H, H10a), 2.33–2.47 (m, 1H, H10b), 2.89–2.99 (m, 1H, H9a), 3.20–3.30 (m, 1H, H9b), 4.37–4.52 (m, 2H, H11), 7.50 (dd, 1H, H6, J = 2.1, 8.9 Hz), 7.65 (d, 2H, H3′,5′, J = 8.7 Hz), 7.75 (d, 1H, H5, J = 8.9 Hz), 7.98–8.01 (m, 3H, H3,2′,6′), 8.17 (d, 1H, H8, J = 2.1 Hz), 9.09 (d, 1H, H2, J = 4.4 Hz). ^13^C NMR (CDCl_3_, 75 MHz) δ ppm: 21.6 (C10), 51.7 (C9), 63.5 (C11), 117.6 (C3), 121.7, 122.7 (C5), 125.6 (q, J = 14.7 Hz), 129.0 (C6), 129.9 (C8), 130.0 (C2′ or 6′), 130.0 (C2′ or 6′), 132.8 (d, J = 4.5 Hz), 134.8 (q, J = 129.9 Hz), 136.5, 148.5, 150.7, 151.3 (C2), 165.1 (C12). Anal. calcd. for: C_20_H_15_ClF_3_NO_3_S: C 54.37, H 4.32, N 3.17; Found: C 54.39, H 4.32, N 3.34. MS: *m*/*z* 442.08. (M+H^+^. 75%).

#### 3.1.4. General Procedure for the Synthesis of Compounds **63**–**82**

A solution of the corresponding sulfanyl benzoate derivative (0.25 mmol) in dry DCM (5 mL) was treated with m-CPBA (2.5 mmol). The mixture was shaken at room temperature (rt) between 8–15 h, under a N_2_ atmosphere. The organic layer was washed with an aqueous mixture of saturated sodium bicarbonate and saturated sodium bisulfite (1:1) (3 × 10 mL), water (2 × 20 mL), a saturated NaCl aqueous solution (2 × 10 mL), and was finally dried over Na_2_SO_4_, filtered, and evaporated under reduced pressure to give the crude product. The compounds were then purified by column chromatography.

2-[(N-Oxide 7-chloroquinolin-4-yl)sulfonyl]ethyl-4-methoxybenzoate (**63**). Column chromathography DCM:EtOAc (9:1). White solid, yield: 61%; m.p. 142–144 °C; IR (KBr) cm^−1^: 3042, 2988, 1709, 1298, 1243, 1137, 1056; ^1^H NMR (CDCl_3_, 300 MHz) δ ppm: 3.80 (t, 2H, H9, J = 5.5 Hz), 3.86 (brs, 3H, OMe), 4.63 (t, 2H, H10, J = 5.3 Hz), 6.76 (d, 2H, H2′,6′, J = 8.9 Hz), 7.31 (d, 2H, H3′,5′, J = 8.9 Hz), 7.69 (dd, 1H, H6, J = 2.2, 9.1 Hz), 7.98 (d, 1H, H3, J = 6.5 Hz), 8.39 (d, 1H, H2, J = 6.5 Hz), 8.52 (d, 1H, H8, J = 2.2 Hz), 8.56 (d, 1H, H5, J = 9.1 Hz). ^13^C NMR (CDCl_3_, 75 MHz) δ ppm: 55.7 (OMe), 56.0 (C9), 57.9 (C10), 113.8 (C3′,5′), 120.3 (C8), 120.4, 124.4, 124.8 (C3), 126.3 (C5), 130.5 (C2′,6′), 131.0 (C6), 132.1, 134.9 (C2), 138.0, 143.0, 164.0, 165.1 (C11). Anal. calcd. for: C_19_H_16_ClNO_6_S: C 54.10, H 3.82, N 3.32; Found: C 54.12, H 3.85, N 3.47. MS: *m*/*z* 422.06. (M+H^+^. 100%).

2-[(N-Oxide 7-chloroquinolin-4-yl)sulfonyl]ethyl-2,3-dimethoxybenzoate (**64**). Column chromathography DCM:EtOAc (9:1). White solid, yield: 51%; m.p. 110 °C; IR (KBr) cm^−1^: 2986, 1725, 1291, 1265, 1145, 1078; ^1^H NMR (CDCl_3_, 300 MHz) δ ppm: 3.68 (brs, 3H, OMe), 3.75 (t, 2H, H9, J = 5.6 Hz), 3.85 (brs, 3H, OMe), 4.61 (t, 2H, H10, J = 5.4 Hz), 6.57 (dd, 1H, H4′, J = 1.5, 7.8 Hz), 6.88 (t, 1H, H5′, J = 7.9 Hz), 7.00 (dd, 1H, H6′, J = 1.4, 8.2 Hz), 7.66 (dd, 1H, H6, J = 2.2, 9.1 Hz), 7.92 (d, 1H, H3, J = 6.5 Hz), 8.34 (d, 1H, H2, J = 6.5 Hz), 8.51 (d, 1H, H8, J = 2.2 Hz), 8.54 (d, 1H, H5, J = 9.1 Hz). ^13^C NMR (CDCl_3_, 75 MHz) δ ppm: 55.8 (C9), 56.1 (OMe), 57.9 (C10), 61.4 (OMe), 116.5 (C4′), 120.1 (C8), 121.1 (C5′), 123.7 (C6′), 123.8, 124.3, 124.8 (C3), 126.2 (C5), 130.4, 131.9 (C6), 134.8 (C2), 137.8, 142.8, 149.1, 153.5, 164.5 (C11). Anal. calcd. for: C_20_H_18_ClNO_7_S: C 53.16, H 4.02, N 3.10; Found: C 53.16, H 4.03, N 3.27. MS: *m*/*z* 452.07. (M+H^+^. 100%).

2-[(N-Oxide 7-chloroquinolin-4-yl)sulfonyl]ethyl-2,5-dimethoxybenzoate (**65**). Column chromathography DCM:EtOAc (9.5:0.5). Yellow solid, yield: 72%; m.p. 133–135 °C; IR (KBr) cm^−1^: 3087, 2991, 1728, 1239, 1227, 1145, 1052; ^1^H NMR (CDCl_3_, 300 MHz) δ ppm: 3.70 (s, 3H, OMe), 3.75 (s, 3H, OMe), 3.79 (t, 2H, H9, J = 5.3 Hz), 4.64 (t, 2H, H10, J = 5.1 Hz), 6.73 (d, 1H, H6′, J = 2.9 Hz), 6.81 (d, 1H, H3′, J = 9.1 Hz), 7.01 (dd, 1H, H4′, J = 2.9, 9.0 Hz), 7.67 (dd, 1H, H6, J = 1.6, 9.1 Hz), 7.95 (d, 1H, H3, J = 6.5 Hz), 8.32 (d, 1H, H2, J = 6.5 Hz), 8.46 (d, 1H, H8, J = 1.5 Hz), 8.56 (d, 1H, H5, J = 9.1 Hz). ^13^C NMR (CDCl_3_, 75 MHz) δ ppm: 55.9 (OMe), 55.9 (C9), 56.3 (OMe), 58.1 (C10), 113.4 (C3′), 116.1 (C6′), 117.7, 119.9 (C4′), 120.1 (C8), 124.4 (C3), 124.8, 126.3 (C5), 130.6, 131.9 (C6), 134.7 (C2), 137.8, 142.8, 152.8, 153.5, 164.4 (C11). Anal. calcd. for: C_20_H_18_ClNO_7_S: C 53.16, H 4.02, N 3.10; Found: C 53.22, H 4.07, N 3.19. MS: *m*/*z* 452.05. (M+H^+^. 100%).

2-[(N-Oxide 7-chloroquinolin-4-yl)sulfonyl]ethyl-2,4,5-trimethoxybenzoate (**66**). Column chromathography DCM:EtOAc (9.5:0.5). Yellow solid, yield: 71%; m.p. 148–150 °C; IR (KBr) cm^−1^: 3080, 2998, 1726, 1295, 1243, 1160, 1025; ^1^H NMR (CDCl_3_, 300 MHz) δ ppm: 3.67 (s, 3H, OMe), 3.74 (s, 3H, OMe), 3.77 (t, 2H, H9, J = 5.3 Hz), 3.94 (s, 3H, OMe), 4.58 (t, 2H, H10, J = 5.1 Hz), 6.35 (s, 1H, H3′), 6.86 (s, 1H, H6′), 7.62 (dd, 1H, H6, J = 2.0, 9.1 Hz), 7.92 (d, 1H, H3, J = 6.5 Hz), 8.32 (d, 1H, H2, J = 6.5 Hz), 8.43 (d, 1H, H8, J = 1.9 Hz), 8.52 (d, 1H, H5, J = 9.1 Hz). ^13^C NMR (CDCl_3_, 75 MHz) δ ppm: 55.9 (C9), 56.2 (OMe), 56.3 (2 × OMe), 57.7 (C10), 96.9 (C3′), 107.9, 113.7 (C6′), 119.9 (C8), 124.3, 124.7 (C3), 126.2 (C5), 130.5, 131.7 (C6), 134.6 (C2), 137.6, 142.4, 142.7, 154.3, 155.6, 164.3 (C11). Anal. calcd. for: C_21_H_20_ClNO_8_S: C 52.34, H 4.18, N 2.91; Found: C 52.36, H 4.17, N 3.12. MS: *m*/*z* 482.06. (M+H^+^. 100%).

2-[(N-Oxide 7-chloroquinolin-4-yl)sulfonyl]ethyl-3,4,5-trimethoxybenzoate (**67**). Column chromathography DCM:EtOAc (9.5:0.5). Yellow solid, yield: 51%; m.p. 168 °C; IR (KBr) cm^−1^: 3017, 2993, 1723, 1294, 1234, 1143, 1036; ^1^H NMR (CDCl_3_, 300 MHz) δ ppm: 3.79–3.83 (m, 8H, 2 × OMe, H9), 3.94 (s, 3H, OMe), 4.65 (t, 2H, H10, J = 5.4 Hz), 6.77 (s, 2H, H2′,6′), 7.69 (dd, 1H, H6, J = 2.2, 9.1 Hz), 7.98 (d, 1H, H3, J = 6.5 Hz), 8.40 (d, 1H, H2, J = 6.5 Hz), 8.50 (d, 1H, H8, J = 2.2 Hz), 8.54 (d, 1H, H5, J = 9.1 Hz). ^13^C NMR (CDCl_3_, 75 MHz) δ ppm: 55.6 (C9), 56.3 (OMe), 58.1 (C10), 61.0 (OMe), 106.4 (C2′,6′), 120.1 (C8), 123.2, 124.3, 124.8 (C3), 126.2 (C5), 130.0, 132.1 (C6), 134.7 (C2), 138.0, 143.0, 153.0, 165.3 (C11). Anal. calcd. for: C_21_H_20_ClNO_8_S: C 52.34, H 4.18, N 2.91; Found: C 52.31, H 4.21, N 3.23. MS: *m*/*z* 482.07. (M+H^+^. 100%).

2-[(N-Oxide 7-chloroquinolin-4-yl)sulfonyl]ethyl-2-methoxybenzoate (**68**). Column chromathography DCM:EtOAc (9.5:0.5). Solid light orange, yield: 72%; m.p. 106–108 °C; IR (KBr) cm^−1^: 3015, 2995, 1731, 1297, 1235, 1142, 1043; ^1^H NMR (CDCl_3_, 300 MHz) δ ppm: 3.73 (s, 3H, OMe), 3.78 (t, 2H, H9, J = 5.5 Hz), 4.62 (t, 2H, H10, J = 5.3 Hz), 6.79 (t, 1H, H3′, J = 7.9 Hz), 6.86 (d, 1H, H4′ or 5′, J = 8.4 Hz), 7.09 (dd, 1H, H4′ or 5′, J = 1.7, 7.8 Hz), 7.44 (td, 1H, H6′, J = 1.8, 7.9 Hz), 7.65 (dd, 1H, H6, J = 2.2, 9.1 Hz), 7.94 (d, 1H, H3, J = 6.5 Hz), 8.31 (d, 1H, H2, J = 6.5 Hz), 8.43 (d, 1H, H8, J = 2.2 Hz), 8.55 (d, 1H, H5, J = 9.1 Hz). ^13^C NMR (CDCl_3_, 75 MHz) δ ppm: 55.8 (OMe), 55.9 (C9), 57.9 (C10), 112.0 (C3′), 117.2, 120.0, 124.4, 124.8 (C3), 126.3 (C5), 130.6 (C6′), 131.1, 131.9 (C6), 134.6 (C4′), 134.7 (C2), 137.8, 142.7, 159.2, 164.4 (C11). Anal. calcd. for: C_19_H_16_ClNO_6_S: C 54.10, H 3.82, N 3.32; Found: C 54.10, H 3.83, N 3.49. MS: *m*/*z* 422.08. (M+H^+^. 100%).

2-[(N-Oxide 7-chloroquinolin-4-yl)sulfonyl]ethyl-4-methoxy-3-nitrobenzoate (**69**). Column chromathography DCM:EtOAc (8:2). Solid light yellow, yield: 53%; m.p. 205–206 °C; IR (KBr) cm^−1^: 3035, 2921, 1724, 1530, 1300, 1238, 1142, 1080; ^1^H NMR (CDCl_3_, 300 MHz) δ ppm: 3.80 (t, 2H, H9, J = 5.6 Hz), 4.06 (s, 3H, OMe), 4.71 (t, 2H, H10, J = 5.4 Hz), 7.04 (d, 1H, H5′, J = 8.9 Hz), 7.65 (dd, 1H, H6′, J = 2.2, 8.8 Hz), 7.75 (dd, 1H, H6, J = 2.3, 9.1 Hz), 8.02 (d, 1H, H3, J = 6.5 Hz), 8.06 (d, 1H, H2′, J = 2.2 Hz), 8.45 (d, 1H, H2, J = 6.5 Hz), 8.56 (d, 1H, H8, J = 2.3 Hz), 8.59 (d, 1H, H5, J = 9.1 Hz). ^13^C NMR (CDCl_3_, 75 MHz) δ ppm: 55.6 (C9), 57.2 (OMe), 58.5 (C10), 113.5 (C5′), 119.6, 120.2 (C8), 120.6, 124.3, 124.9 (C3), 126.3 (C5), 127.0 (C2′), 128.8, 132.4 (C2), 134.7 (C2′),134.9, 138.4, 143.1, 156.9, 163.4 (C11). Anal. calcd. for: C_19_H_15_ClN_2_O_8_S: C 48.88, H 3.24, N 6.00; Found: C 48.89, H 3.27, N 5.89. MS: *m*/*z* 467.05. (M+H^+^. 100%).

2-[(N-Oxide 7-chloroquinolin-4-yl)sulfonyl]ethyl-5-methyl-2-nitrobenzoate (**70**). Column chromathography DCM:EtOAc (9.5:0.5). Yellow solid, yield: 48%; m.p. 148–150 °C; IR (KBr) cm^−1^: 2983, 1743, 1523, 1341, 1297, 1139; ^1^H NMR (CDCl_3_, 300 MHz) δ ppm: 2.45 (s, 3H, CH_3_), 3.71 (t, 2H, H9, J = 5.6 Hz), 4.68 (t, 2H, H10, J = 5.4 Hz), 7.06 (s, 1H, H6′), 7.39 (d, 1H, H4′, J = 8.3 Hz), 7.69 (dd, 1H, H6, J = 2.0, 9.1 Hz), 7.75 (d, 1H, H3′, J = 8.3 Hz), 7.94 (d, 1H, H3, J = 6.5 Hz), 8.38 (d, 1H, H2, J = 6.5 Hz), 8.52 (d, 1H, H8, J = 2.0 Hz), 8.58 (d, 1H, H5, J = 9.1 Hz). ^13^C NMR (CDCl_3_, 75 MHz) δ ppm: 21.5 (CH_3_), 55.5 (C9), 59.0 (C10), 120.0 (C8), 124.3, 124.4, 124.9, 126.4, 126.5, 129.5, 130.3, 132.1, 132.6, 134.8, 138.1, 142.7, 145.0, 145.4, 164.8 (C11). Anal. calcd. for: C_19_H_15_ClN_2_O_7_S: C 50.62, H 3.35, N 6.21; Found: C 50.65, H 3.34, N 6.47. MS: *m*/*z* 451.03. (M+H^+^. 100%).

2-[(N-Oxide 7-chloroquinolin-4-yl)sulfonyl]ethyl-,5-dimethylbenzoate (**71**). Column chromathography DCM:EtOAc (8:2). White solid, yield: 70%; m.p. 172–174 °C; IR (KBr) cm^−1^: 2931, 1720, 1275, 1158, 1025; ^1^H NMR (CDCl_3_, 300 MHz) δ ppm: 2.27 (s, 6H, 2 × CH_3_), 3.83 (t, 2H, H9, J = 5.4 Hz), 4.66 (t, 2H, H10, J = 5.3 Hz), 6.97 (s, 2H, H2′,6′), 7.15 (s, 1H, H4′), 7.68 (dd, 1H, H6, J = 2.2, 9.12 Hz), 7.98 (d, 1H, H3, J = 6.5 Hz), 8.38 (d, 1H, H2, J = 6.5 Hz), 8.48 (d, 1H, H8, J = 2.2 Hz), 8.55 (d, 1H, H5, J = 9.1 Hz). ^13^C NMR (CDCl_3_, 75 MHz) δ ppm: 21.2 (2 × CH_3_), 55.9 (C9), 58.2 (C10), 120.2 (C8), 124.3, 124.8 (C3), 126.2 (C5), 126.6 (C2′,6′), 128.0, 130.5, 132.1 (C6), 134.7, 135.5 (C2), 138.0, 138.5, 142.9, 165.8 (C11). Anal. calcd. for: C_20_H_18_ClNO_5_S: C 57.21, H 4.32, N 3.34; Found: C 57.18, H 4.35, N 3.51. MS: *m*/*z* 420.07. (M+H^+^. 100%).

2-[(N-Oxide 7-chloroquinolin-4-yl)sulfonyl]ethyl-4-(trifluoromethyl)benzoate (**72**). Column chromathography DCM:EtOAc (8:2). White solid, yield: 73%; m.p. 115–117 °C; IR (KBr) cm^−1^: 3029, 2922, 1731, 1318, 1265, 1239, 1069; ^1^H NMR (CDCl_3_, 300 MHz) δ ppm: 3.79 (t, 2H, H9, J = 5.6 Hz), 4.72 (t, 2H, H10, J = 5.4 Hz), 7.59–7.66 (m, 4H, H2′,3′,5′,6′), 7.70 (dd, 1H, H6, J = 2.3, 9.1 Hz), 8.01 (d, 1H, H3, J = 6.5 Hz), 8.46 (d, 1H, H2, J = 6.5 Hz), 8.54 (d, 1H, H8, J = 2.2 Hz), 8.58 (d, 1H, H5, J = 9.1 Hz). ^13^C NMR (CDCl_3_, 75 MHz) δ ppm: 55.7 (C9), 58.5 (C10), 120.3 (C8), 124.3, 124.9 (C3), 125.6 (q, J = 14.6 Hz), 126.2 (C5), 129.6 (C2′,6′), 129.9, 130.5, 131.6 (d, J = 4.4 Hz), 132.3 (C6), 134.9 (C2), 135.2 (q, J = 130.7 Hz), 138.3, 143.0, 151.1, 164.4 (C11). 19F NMR (CDCl_3_) δ ppm: −63.24. Anal. calcd. for: C_19_H_13_ClF_3_NO_5_S: C 49.63, H 2.85, N 3.05; Found: C 49.65, H 2.87, N 3.27. MS: *m*/*z* 460.12. (M+H^+^. 78%).

3-[(N-Oxide 7-chloroquinolin-4-yl)sulfonyl]propyl-4-methoxybenzoate (**73**). Column chromathography DCM:EtOAc (8:2). White solid, yield: 64%; m.p. 135–137 °C; IR (KBr) cm^−1^: 3057, 2979, 1699, 1310, 1211, 1146, 1084; ^1^H NMR (CDCl_3_, 300 MHz) δ ppm: 2.16–2.25 (m, 2H, H10), 3.43 (t, 2H, H9, J = 7.6 Hz), 3.85 (s, 3H, OMe), 4.31 (t, 2H, H11, J = 6.0 Hz), 6.85 (d, 2H, H3′,5′, J = 8.85 Hz), 7.68 (d, 1H, H6, J = 2.1, 9.1 Hz), 7.75 (d, 2H, H2′,6′, J = 8.9 Hz), 7.99 (d, 1H, H3, J = 6.5 Hz), 8.53 (d, 1H, H2, J = 6.5 Hz), 8.59 (d, 1H, H5, J = 9.1 Hz), 8.71 (d, 1H, H8, J = 2.1 Hz). ^13^C NMR (CDCl_3_, 75 MHz) δ ppm: 22.7 (C10), 53.4 (C9), 55.6 (OMe), 61.7 (C11), 113.8 (C3′,5′), 120.4 (C8), 121.6, 124.4, 124.9 (C3), 126.4 (C5), 129.7, 131.5 (C2′,6′). 132.1 (C6), 134.9 (C2), 138.2, 143.0, 163.8, 165.8 (C12). Anal. calcd. for: C_20_H_18_ClNO_6_S: C 55.11, H 4.16, N 3.21; Found: C 55.10, H 4.16, N 3.41. MS: *m*/*z* 436.08. (M+H^+^. 100%).

3-[( N-Oxide 7-chloroquinolin-4-yl)sulfonyl]propyl-2,3-dimethoxybenzoate (**74**). Column chromathography DCM:EtOAc (9:1). Cream solid, yield: 75%; m.p. 106–108 °C; IR (KBr) cm^−1^: 3059, 2939, 1704, 1305, 1235, 1149, 1043; ^1^H NMR (CDCl_3_, 300 MHz) δ ppm: 2.18–2.27 (m, 2H, H10), 3.45 (t, 2H, H9, J = 7.6 Hz), 3.77 (s, 3H, OMe), 3.87 (s, 3H, OMe), 4.36 (t, 2H, H11, J = 5.9 Hz), 7.02–7.05 (m, 2H, H4′,5′), 7.13 (dd, 1H, H6′, J = 3.4, 6.18 Hz), 7.69 (dd, 1H, H6, J = 2.16, 9.09 Hz), 7.97 (d, 1H, H3, J = 6.48 Hz), 8.51 (d, 1H, H2, J = 6.5 Hz), 8.60 (d, 1H, H5, J = 9.1 Hz), 8.71 (d, 1H, H8, J = 2.2 Hz). ^13^C NMR (CDCl_3_, 75 MHz) δ ppm: 22.5 (C10), 53.3 (C9), 56.1 (OMe), 61.4 (OMe), 62.1 (C11), 116.1 (C4′), 120.2 (C8), 122.0 (C5′), 124.0 (C6′), 124.3, 124.7 (C3), 125.3, 126.6 (C5), 129.9, 132.1 (C6), 134.9 (C2), 138.2, 143.0, 149.0, 153.5, 165.9 (C12). Anal. calcd. for: C_21_H_20_ClNO_7_S: C 54.14, H 4.33, N 3.01; Found: C 54.12, H 4.32, N 3.27. MS: *m*/*z* 466.09. (M+H^+^. 100%).

3-[( N-Oxide 7-chloroquinolin-4-yl)sulfonyl]propyl-2,5-dimethoxybenzoate (**75**). Column chromathographyc DCM:EtOAc (9:1). Cream solid, yield: 65%; m.p. 127–128 °C; IR (KBr) cm^−1^: 3058, 2968, 1712, 1300, 1214, 1144, 1027; ^1^H NMR (CDCl_3_, 300 MHz) δ ppm: 2.15–2.24 (m, 2H, H10), 3.46 (t, 2H, H9, J = 6.7 Hz), 3.75 (s, 6H, OMe), 4.33 (t, 2H, H11, J = 6.6 Hz), 6.86 (d, 1H, H3′, J = 9.1 Hz), 6.99 (dd, 1H, H4′, J = 3.2, 9.1 Hz), 7.15 (d, 1H, H6′, J = 3.2 Hz), 7.67 (dd, 1H, H6, J = 2.2, 9.1 Hz), 7.97 (d, 1H, H3, J = 6.5 Hz), 8.51 (d, 1H, H2, J = 6.5 Hz), 8.60 (d, 1H, H5, J = 9.1 Hz), 8.69 (d, 1H, H8, J = 2.2 Hz). ^13^C NMR (CDCl_3_, 75 MHz) δ ppm: 22.6 (C10), 53.4 (C9), 55.9 (OMe), 56.6 (OMe), 62.0 (C11), 113.7, 116.3, 119.6, 120.3 (C8), 124.3 (C4′), 124.8 (C3), 126.5 (C5), 129.9, 132.0 (C6), 134.9 (C2), 138.2, 143.0, 153.0, 153.5, 165.6 (C12). Anal. calcd. for: C_21_H_20_ClNO_7_S: C 54.14, H 4.33, N 3.01; Found: C 54.17, H 4.28, N 3.19. MS: *m*/*z* 466.11. (M+H^+^. 100%).

3-[(N-Oxide 7-chloroquinolin-4-yl)sulfonyl]propyl-2,4,5-trimethoxybenzoate (**76**). Column chromathography DCM:EtOAc (9:1). Yellow solid, yield: 61%; m.p. 148 °C; IR (KBr) cm^−1^: 3054, 2931, 1679, 1314, 1245, 1209, 1041; ^1^H NMR (CDCl_3_, 300 MHz) δ ppm: 2.14–2.23 (m, 2H, H10), 3.46 (t, 2H, H9, J = 7.6 Hz), 3.77 (s, 3H, OMe), 3.81 (s, 3H, OMe), 3.92 (s, 3H, OMe), 4.29 (t, 2H, H11, J = 5.9 Hz), 6.44 (s, 1H, H3′), 7.22 (s, 1H, H6′), 7.65 (dd, 1H, H6, J = 2.2, 9.1 Hz), 7.97 (d, 1H, H3, J = 6.5 Hz), 8.51 (d, 1H, H2, J = 6.5 Hz), 8.59 (d, 1H, H5, J = 9.1 Hz), 8.68 (d, 1H, H8, J = 2.2 Hz). ^13^C NMR (CDCl_3_, 75 MHz) δ ppm: 22.7 (C10), 53.5 (C9), 56.1 (OMe), 56.5 (OMe), 56.8 (OMe), 61.6 (C11), 97.4 (C3′), 109.5, 114.4 (C6′), 120.2 (C8), 124.3, 124.8 (C3), 126.5 (C5), 129.8, 131.9 (C6), 134.9 (C2), 138.1, 142.6, 143.0, 154.1, 155.7, 165.3 (C12). Anal. calcd. for: C_22_H_22_ClNO_8_S: C 53.28, H 4.47, N 2.82; Found: C 53.28, H 4.48, N 3.07. MS: *m*/*z* 496.10. (M+H^+^. 100%).

3-[(N-Oxide 7-chloroquinolin-4-yl)sulfonyl]propyl-3,4,5-trimethoxybenzoate (**77**). Column chromathography DCM:EtOAc (9:1). White solid, yield: 64%; m.p. 187–188 °C; IR (KBr) cm^−1^: 3056, 2927, 1708, 1314, 1213, 1147, 1027; ^1^H NMR (CDCl_3_, 300 MHz) δ ppm: 2.17–2.26 (m, 2H, H10), 3.40 (t, 2H, H9, J = 7.5 Hz), 3.83 (s, 6H, 2 × OMe), 3.87 (s, 3H, OMe), 4.34 (t, 2H, H11, J = 6.1 Hz), 7.10 (s, 2H, H2′,6′), 7.65 (dd, 1H, H6, J = 2.2, 9.1 Hz), 7.97 (d, 1H, H3, J = 6.5 Hz), 8.51 (d, 1H, H2, J = 6.5 Hz), 8.58 (d, 1H, H5, J = 9.1 Hz), 8.67 (d, 1H, H8, J = 2.2 Hz). ^13^C NMR (CDCl_3_, 75 MHz) δ ppm: 22.6 (C10), 53.3 (C9), 56.3 (2 × OMe), 61.0 (OMe), 62.3 (C11), 106,8 (C2′,6′), 120.3 (C8), 124.2, 124.3, 124.9 (C3), 126.3 (C5), 129.6, 132.0 (C6), 134.9 (C2), 138.2, 142.6, 143.0, 153.0, 165.7 (C12). Anal. calcd. for: C_22_H_22_ClNO_8_S: C 53.28, H 4.47, N 2.82; Found: C 53.30, H 4.45, N 2.98. MS: *m*/*z* 496.09. (M+H^+^. 100%).

3-[(N-Oxide 7-chloroquinolin-4-yl)sulfonyl]propyl-2-methoxybenzoate (**78**). Column chromathography DCM:EtOAc (9.5:0.5). Solid light orange, yield: 67%; m.p. 106–108 °C; IR (KBr) cm^−1^: 3053, 2933, 1718, 1305, 1212, 1145, 1020; ^1^H NMR (CDCl_3_, 300 MHz) δ ppm: 2.13–2.22 (m, 2H, H10), 3.45 (t, 2H, H9, J = 7.7 Hz), 3.80 (s, 3H, OMe), 4.31 (t, 2H, H11, J = 5.9 Hz), 6.87–6.93 (m, 2H, H3′,5′), 7.41–7.47 (td, 1H, H4′, J = 1.8, 8.6 Hz), 7.58 (dd, 1H, H6′, J = 1.8, 7.6 Hz), 7.66 (dd, 1H, H6, J = 2.2, 9.1 Hz), 7.97 (d, 1H, H3, J = 6.5 Hz), 8.51 (d, 1H, H2, J = 6.5 Hz), 8.60 (d, 1H, H5, J = 9.1 Hz), 8.69 (d, 1H, H8, J = 2.2 Hz). ^13^C NMR (CDCl_3_, 75 MHz) δ ppm: 22.6 (C10), 53.4 (C9), 55.9 (OMe), 61.8 (C11), 112.1 (C3′), 119.1, 120.2 (C5′ or 8), 120.3 (C5′ or 8), 124.3, 124.8 (C3), 126.5 (C5), 129.8, 131.5 (C6′), 131.9 (C6), 134.0 (C4′), 134.9 (C2), 138.1, 142.9, 159.2, 165.7 (C12). Anal. calcd. for: C_20_H_18_ClNO_6_S: C 55.11, H 4.16, N 3.21; Found: C 55.10, H 4.15, N 3.39. MS: *m*/*z* 436.07. (M+H^+^. 100%).

3-[(N-Oxide 7-chloroquinolin-4-yl)sulfonyl]propyl-4-methoxy-3-nitrobenzoate (**79**). Column chromathographyc DCM:EtOAc (9:1). Solid light yellow, yield: 63%; m.p. 205–206 °C; IR (KBr) cm^−1^: 3038, 2927, 1706, 1530, 1364, 1239, 1144, 1079; ^1^H NMR (CDCl_3_, 300 MHz) δ ppm: 2.23–2.32 (m, 2H, H10), 3.43 (t, 2H, H9, J = 7.5 Hz), 4.04 (s, 3H, OMe), 4.40 (t, 2H, H11, J = 6.2 Hz), 7.11 (d, 1H, H5′, J = 8.9 Hz), 7.75 (dd, 1H, H6, J = 2.2, 9.1 Hz), 8.02 (d, 1H, H3, J = 6.5 Hz), 8.06 (dd, 1H, H6′, J = 2.2, 8.8 Hz), 8.33 (d, 1H, H2′, J = 2.2 Hz), 8.55 (d, 1H, H2, J = 6.5 Hz), 8.64 (d, 1H, H5, J = 9.1 Hz), 8.75 (d, 1H, H8, J = 2.2 Hz). ^13^C NMR (CDCl_3_, 75 MHz) δ ppm: 22.6 (C10), 53.3 (C9), 57.1 (OMe), 62.7 (C11), 113.4 (C5′), 120.5 (C8), 121.7, 124.4, 125.0 (C3), 126.4 (C5), 127.2 (C2′), 129.7, 132.3 (C6), 135.0 (C2), 135.4 (C6′), 138.4, 156.6, 164.1 (C12). Anal. calcd. for: C_20_H_17_ClN_2_O_8_S: C 49.95, H 3.56, N 5.83; Found: C 49.97, H 3.56, N 5.95. MS: *m*/*z* 481.06. (M+H^+^. 83%).

3-[(N-Oxide 7-chloroquinolin-4-yl)sulfonyl]propyl-5-methyl-2-nitrobenzoate (**80**). Column chromathography DCM:EtOAc (9:1). White solid, yield: 60%; m.p. 182–184 °C; IR (KBr) cm^−1^: 3021, 2971, 1740, 1512, 1341, 1203, 1037; ^1^H NMR (CDCl_3_, 300 MHz) δ ppm: 2.19–2.28 (m, 2H, H10), 2.46 (s, 3H, CH_3_), 3.40 (t, 2H, H9, J = 7.6 Hz), 4.42 (t, 2H, H11, J = 5.8 Hz), 7.39–7.41 (m, 2H, H 4′,6′), 7.76 (dd, 1H, H6, J = 2.3, 9.2 Hz), 7.80 (d, 1H, H3′, J = 8.8 Hz), 8.01 (d, 1H, H3, J = 6.5 Hz), 8.55 (d, 1H, H2, J = 6.5 Hz), 8.68 (d, 1H, H5, J = 9.1 Hz), 8.77 (d, 1H, H8, J = 2.2 Hz). ^13^C NMR (CDCl_3_, 75 MHz) δ ppm: 21.5 (CH_3_), 22.2 (C10), 53.5 (C9), 63.7 (C11), 120.3 (C8), 124.2 (C3′), 124.5 (C3), 124.8, 126.8 (C5), 127.5, 130.1, 130.3 (C6′), 132.2 (C6), 132.4 (C4′), 134.9 (C2), 138.3, 143.1, 145.0, 145.6, 165.6 (C12). Anal. calcd. for: C_20_H_17_ClN_2_O_7_S: C 51.67, H 3.69, N 6.03; Found: C 51.69, H 3.66, N 6.23. MS: *m*/*z* 465.07. (M+H^+^. 97%).

3-[(N-Oxide 7-chloroquinolin-4-yl)sulfonyl]propyl-3,5-dimethylbenzoate (**81**). Column chromathography DCM:EtOAc (9:1). White solid, yield: 66%; m.p. 188–190 °C; IR (KBr) cm^−1^: 3019, 2915, 1707, 1363, 1290, 1043; ^1^H NMR (CDCl_3_, 300 MHz) δ ppm: 2.19–2.28 (m, 2H, H10), 2.32 (s, 6H, 2 × CH_3_), 3.44 (t, 2H, H9, J = 7.6 Hz), 4.34 (t, 2H, H11, J = 6.0 Hz), 7.18 (brs, 1H, H4′), 7.45 (s, 2H, H2′, 6′), 7.66 (dd, 1H, H6, J = 2.2, 9.1 Hz), 7.99 (d, 1H, H3, J = 6.5 Hz), 8.53 (d, 1H, H2, J = 6.5 Hz), 8.61 (d, 1H, H5, J = 9.1 Hz), 8.70 (d, 1H, H8, J = 2.2 Hz). ^13^C NMR (CDCl_3_, 75 MHz) δ ppm: 21.3 (2 × CH_3_), 22.7 (C10), 53.4 (C9), 61.9 (C11), 120.4 (C8), 124.4, 124.9 (C3), 126.4 (C5), 127.2 (C2′,6′), 129.2, 129.7, 132.1 (C6), 134.9, 135.2 (C2), 138.2, 138.3, 143.1, 166.4 (C12). Anal. calcd. for: C_21_H_20_ClNO_5_S: C 58.13, H 4.65, N 3.23; Found: C 58.19, H 4.69, N 3.28. MS: *m*/*z* 434.12. (M+H^+^. 100%).

3-[(N-Oxide 7-chloroquinolin-4-yl)sulfonyl]propyl-4-(trifluoromethyl)benzoate (**82**). Column chromathography DCM:EtOAc (7:3). White solid, yield: 58%; m.p. 139–140 °C; IR (KBr) cm^−1^: 3021, 2910, 1718, 1320, 1283, 1151, 1022; ^1^H NMR (CDCl_3_, 300 MHz) δ ppm: 2.21–2.30 (m, 2H, H10), 3.43 (t, 2H, H9, J = 7.4 Hz), 4.41 (t, 2H, H11, J = 5.9 Hz), 7.64–7.71 (m, 3H, H3′,5′,6), 7.98 (d, 2H, H2′,6′, J = 8.4 Hz), 8.00 (d, 1H, H3, J = 6.9 Hz), 8.54 (d, 1H, H2, J = 6.9 Hz), 8.61 (d, 1H, H5, J = 9.1 Hz), 8.71 (d, 1H, H8, J = 1.2 Hz). ^13^C NMR (CDCl_3_, 75 MHz) δ ppm: 22.6 (C10), 53.3 (C9), 62.7 (C11), 120.4 (C8), 124.3, 125.0 (C3), 125.6 (q, J = 14.4 Hz), 126.4 (C5), 129.6, 130.0 (C2′,6′), 132.1 (C6), 132.6 (d, J = 3.8 Hz), 134.9 (q, J =126.1 Hz), 138.3, 143.1, 164.9 (C12). 19F NMR (CDCl_3_) δ ppm: −63.14. Anal. calcd. for: C_20_H_15_ClF_3_NO_5_S: C 50.69, H 3.19, N 2.96; Found: C 50.69, H 3.21, N 3.25. MS: *m*/*z* 474.07. (M+H^+^. 78%).

### 3.2. X-ray Analysis on Compound **15**

Colourless blocky crystals of compound **15** were grown from the slow evaporation of an ethanol solution. Single crystal X-ray diffraction analyses were carried out on a Bruker APEX Kappa Duo Diffractometer Mo-Kα radiation (λ = 0.71073Å), and a graphite monochromator. Data collection and unit cell refinement were carried out with SMART [42] and data reduction with SAINT [43]. The structure was solved by direct methods and refined by least-squares techniques with SHELXS and SHELXL [44], respectively, using OLEX2 [45] as an interface. Hydrogen atoms were placed in calculated positions and refined using a riding model with their displacement parameters equal to 1.2 Uiso of the non-hydrogen atom to which they are attached.

X-ray crystallographic data for this structure has been deposited at the Cambridge Crystallographic Data Center under code CCDC 2184667. Copies of the data can be obtained free of charge from The Cambridge Crystallographic Data Centre via www.ccdc.cam.ac.uk/structures (accessed on 7 July 2022).

### 3.3. Biological Activity

#### 3.3.1. Cell Lines

All cell lines were purchased from the American Tissue Culture Collection (ATCC). The A549 cell line is lung adenocarcinoma. MRC-5 and BJ cell lines were used as a non-tumor control and represent human fibroblasts. (MRC-5 LD and BJLD) are doxorubicin-resistant human lung fibroblast cell lines. The CCRF-CEM line is derived from T lymphoblastic leukemia, evincing high chemosensitivity, whereas K562 represent cells from an acute myeloid leukemia patient sample with bcr-abl translocation. The daunorubicin-resistant subline of CCRF-CEM cells (CEM-DNR bulk) and paclitaxel-resistant subline K562-TAX were selected in our laboratory by the cultivation of maternal cell lines in increasing concentrations of daunorubicin or paclitaxel, respectively. The CEM-DNR bulk cells overexpress MRP-1 and P-glycoprotein protein, whereas K562-TAX cells overexpress P-glycoprotein only. Both proteins belong to the family of ABC transporters and are involved in the primary and/or acquired multidrug-resistance phenomenon. The U2OS cell line is derived from osteosarcoma, HCT116 is a colorectal tumor cell line and its p53 gene knock-down counterpart (HCT116p53−/−, Horizon Discovery Ltd., Cambridge, UK) is a model of human cancers with p53 mutation frequently associated with poor prognosis [33,36]. The cells were maintained in nunc/corning 80 cm^2^ plastic tissue culture flasks and cultured in a cell culture medium according to ATCC or Horizon recommendations (DMEM/RPMI 1640 with 5 g/L glucose, 2 mM glutamine, 100 U/mL penicillin, 100 mg/mL streptomycin, 10% fetal calf serum, and NaHCO_3_).

#### 3.3.2. Cytotoxic MTS Assay 

The activity of compounds was determined using a standard 3-(4,5-dimethylthiazol-2-yl)-5-(3-carboxymethoxyphenyl)-2-(4-sulfophenyl)-2H-tetrazolium (MTS) and it was performed at the Institute of Molecular and Translational Medicine by a robotic platform (High-ResBiosolutions). Cell suspensions were prepared and diluted according to the particular cell type and the expected target cell density (25,000–35,000 cells/mL based on cell growth characteristics). Cells were added by automatic pipettor (30 µL) into 384-well microtiter plates. All tested compounds were dissolved in 100% DMSO and four-fold dilutions of the intended test concentration were added in 0.15 µL aliquots at time zero to the microtiter plate wells by the echo acoustic non-contact liquid handler Echo550 (Labcyte). The experiments were performed in technical duplicates and three biological replicates at least. The cells were incubated with the tested compounds for 72 h at 37 °C, in a 5% CO_2_ atmosphere at 100% humidity. At the end of the incubation period, the cells were assayed by using the MTS test. Aliquots (5 µL) of the MTS stock solution were pipetted into each well and incubated for an additional 1-4 h. After this incubation period, the optical density (OD) was measured at 490 nm with an Envision reader (PerkinElmer). Tumor cell survival (TCS) was calculated by using the following equation: TCS = (ODdrug-exposed well/mean ODcontrol wells) × 100%. The IC_50_ value, the compound concentration that is lethal to 50% of the tumor cells, was calculated from the appropriate dose-response curves in Dotmatics software (Updated version 2022, London, UK) [33,34,35,36].

#### 3.3.3. Cell Cycle and Apoptosis Analysis 

CCRF-CEM cells were seeded at a density of 1 × 10^6^ cells per one mL in 6-well plates (TTP) and were cultivated with compounds at concentrations corresponding to 1 × or 5 × IC_50_ value. Together with the compounds-treated cells, a vehicle-treated sample was harvested at the same time point. After 24 h, the cells were washed with cold phosphate-buffered saline (PBS) and fixed in 70% ethanol added dropwise and stored overnight at −20 °C. Afterward, cells were washed in hypotonic citrate buffer, treated with RNase (50 µg mL^−1^), and stained with propidium iodide. Flow cytometry using a 488 nm single beam laser (Becton Dickinson) was used for measurement. The cell cycle was analyzed by the software ModFitLT (Verity), and apoptosis was measured in a logarithmic model expressing the percentage of the particles with propidium content lower than cells in the G0/G1 phase (<G1) of the cell cycle. Half of the sample was used for pH3Ser10 antibody (Sigma) labeling and subsequent flow cytometry analysis of the cells in mitosis [35].

#### 3.3.4. BrDU Incorporation Analysis 

Cells were cultivated and processed as described in the previous method [33,34,35,36]. Before harvesting, BrDU 10 µM was added to the cells for pulse-labeling for 30 min. Then, cells were washed by PBS and fixed with −20 °C cold 70% ethanol and stored in a freezer overnight. Before analysis, the samples were incubated on ice for 30 min, washed once with PBS, and re-suspended in 2 M HCl for 30 min at rt to denature their DNA. Following neutralization with a 0.1 M Na_2_B_4_O_7_ (borax) solution, the cells were washed with PBS containing 0.5% Tween-20 and 1% BSA. Next, staining with primary anti-BrDU antibody (Exbio) was performed for 30 min at rt in the dark. Cells were then washed with PBS and stained with secondary anti-mouse-FITC antibody (Sigma) at rt in the dark. After another wash with PBS and incubation with propidium iodide (0.1 mg × mL^−1^) and RNase A (0.5 mg × mL^−1^) for 1 h at rt in the dark, cells were analyzed by flow cytometry using a 488 nm single beam laser (FACSCalibur, Becton Dickinson, Franklin Lakes, NJ, USA).

#### 3.3.5. BrU Incorporation Analysis 

Cells were cultured and treated as described above [33,34,35,36]. Before harvesting, pulse-labeling with 1 mM BrU for 30 min followed. The cells were then fixed in 1% buffered paraformaldehyde with 0.05% NP-40 at rt for 15 min, and then stored at 4 °C overnight. Before measurement, they were washed with 1% glycine in PBS, washed with PBS again and stained with primary anti-BrDU antibody cross-reacting to BrU (Exbio) for 30 min at rt in the dark. From this point, the experiment was performed exactly as in the previous method.

## 4. Conclusions

In this work, we described a convenient and efficient method for the synthesis of a series of [(7-chloroquinolin-4-yl)thio]alkyl benzoate derivatives **5**–**82** through the reaction of [(7-chloroquinolin-4-yl)thio]alcohols **3** and **4** with different benzoic acids, under a modified version of the Steglich esterification reaction. To obtain sulfinyl derivatives **41**–**62** and sulfonyl analogues **63**–**82**, m-CPBA was used as an oxidizing agent. By modifying the degree of oxidation of both the sulfur atom and the quinolinic nitrogen, and by varying the length of the alkyl spacer that binds the head group and the benzoic acids, we fine-tuned the selectivity and potency of our compounds as inhibitors of a series of cancer cells. The most active were the 3-[(N-oxide 7-chloroquinolin-4-yl)sulfonyl]propyl benzoate derivatives, which exert micromolar cytotoxic effects against cells derived from lung and colorectal carcinoma. In some cases, compounds such as **73**, **74**, and **81** also show good selectivity over proliferating cancer cell lines with low toxicity to non-malignant MRC or BJ fibroblasts. A low cytotoxicity was observed against multidrug-resistant cancer cell lines (CEM-DNR, K562-TAX), suggesting that they are substrates for pharmacological transporters. At higher concentrations (5 × IC_50_) against the CCRF-CEM cancer cell line, we observed the accumulation of the cells in the G0/G1 cell phase, inhibition of DNA and RNA synthesis, and induction of apoptosis. The molecular mechanism of this latter effect requires additional investigation. It is known that the lipophilicity of the compounds plays an important role in their penetration into cells and in cell permeability, but this dependence was not clear for all tested cancer cell lines.

The results presented in this study reveal that these kinds of compounds are highly selective inhibitors of cancer cell lines in vitro and thus show potential for further development of antitumor agents.

## Figures and Tables

**Table 1:** Crystal data and refinement for compound **15**.

Crystal Data	
**Formula**	C_18_H_13_Cl_2_NO_2_S
**Formula Weight**	378.25
**Crystal System**	Triclinic
**Space group**	$P\bar{1}$(No. 2)
**a, b, c (Å)**	7.9509 (7), 9.8444 (8), 11.6086 (10)
**α, β, γ (º)**	81.312 (4), 89.355 (4), 69.186 (4)
**V (Å^3^)**	838.70 (13)
**Z**	2
**D(calc) (g/cm^3^)**	1.498
**Mu (MoKα) (/mm)**	0.522
**F (000)**	388
**Crystal Size (mm)**	0.01 × 0.02 × 0.04
**Data Collection**	
**Temperature (K)**	296
**Radiation (Å)**	MoKα 0.71073
**Theta Min-Max (º)**	1.8, 28.5
**Dataset**	−10:10;−13:12;−15:14
**Tot., Uniq. Data, R (int)**	15,344, 4252, 0.023
**Observed Data [I > 0.0sigma (I)]**	3189
**Refinement**	
**Nref, Npar**	4252, 217
**R, wR2, S**	0.0373, 0.1117, 1.04
**Max. and Av. Shift/Error**	0.00, 0.00
**Min. and Max. Resd. Dens. (e/Ang^3^)**	−0.28, 0.35

**Table 2:** Hydrogen bonds and Y-X…Cg interaction for compounds **15**.

Donor—H…Acceptor	D-H (Å)	H…A (Å)	D…A (Å)	D-H…A (^o^)	Graph
C_10_-H_10_…O_1_	0.9300	2.2900	2.637 (2)	102.00	S(5)
C_14_-H_14_…S_1_	0.9300	2.6300	3.0400 (18)	107.00	S(5)
**Y-X…Cg**	**X..Cg (Å)**	**Y-X..Cg (^o^)**	**Y..Cg (Å)**	**Symmetry**	
C_1_-Cl_1_…Cg_3_	3.9111 (10)	98.18 (6)	4.5004 (19)	X, Y, 1 + Z	

C_g3†_ C9-10-C11-C12-C18-C17.

**Table 3:** Cytotoxic activity (IC_50_, µM) of 7-chloro-(4-thioalkylquinoline) derivatives **41**–**82** after 72 h of incubation with cancerous and noncancerous human cell lines.

No	A549	BJ	BJLD	CCRF- CEM	CEM- DNR	HCT116	HCT116- p53	K562	K562- TAX	MRC-5	MRC5-LD	U2OS
**41**	34.42	33.28	28.54	4.52	35.07	23.52	22.47	27.8	21.37	26.5	24.52	22.11
**47**	32.18	31.17	30.73	2.74	26.84	15.91	11.34	16.87	11.26	28.24	26.09	19.08
**48**	25.04	29.19	27.53	1.42	12.88	16.99	10.04	16.89	9.03	27.82	23.97	18.58
**49**	15.65	30.74	23.49	1.34	7.75	7	7.56	12.29	6.4	>50	>50	11.22
**50**	23.98	30.83	28.54	1.32	10.03	7.99	7.1	15.2	9.58	26.86	22.34	8.57
**53**	23.57	30.01	28.22	1.31	8.71	12.53	8.51	13.91	6.98	27.89	22.42	12.82
**54**	25.79	30.43	26.86	1.09	10.14	14.03	9.19	14.12	7.63	27.18	25.05	17.17
**57**	30.49	33.02	31.88	1.51	16.01	19.29	14.79	17.44	14.56	29.92	25.81	21.32
**59**	7.76	21.6	10.23	1.2	8.81	8.86	6.81	8.77	6.12	23.68	10.33	5.15
**60**	9.55	28.96	18.1	1.13	7.27	7.77	6.06	8.86	4.26	29.35	19.82	5.81
**61**	10.92	30.32	27.64	1.05	8.35	8.26	6.89	12.66	6.76	26.09	22.06	8.1
**62**	26.12	29.91	30.1	1.17	10.53	15.66	9.61	17.63	9.15	29.35	25.85	14.29
**63**	28.81	23.93	12.06	0.77	8.74	16.23	16.39	5.38	4.71	4	2.91	5.71
**64**	28.42	9.19	8.95	0.71	8.99	8.18	7.68	5.51	5.65	6.08	3.28	7.43
**65**	28.22	22.2	9.68	0.55	8.97	8.83	8.26	5.85	6.65	4.95	2.02	7.67
**66**	28.01	21.22	10.95	1.11	8.03	9.22	7.94	5.3	6.25	5.07	2.3	8.25
**67**	28.36	24.8	11.86	1.05	8.93	11.08	7.87	6.05	5.19	5.79	3.24	9.23
**68**	27.04	24.6	10.08	1.23	12.2	5.25	8.3	4.72	4.63	4.92	4.17	6.9
**69**	28.26	27.22	19.53	1.38	19.08	13.12	11.22	6.49	7.32	8.41	2.74	10.3
**70**	28.25	25.67	>50	1.16	17.43	11.49	8.75	6.1	6.89	22.9	6.47	9.92
**72**	28.49	28.73	26.96	2.14	25.2	17.31	16.43	3.02	6.06	24.43	13.9	18.37
**73**	5.44	12.4	10.66	2.18	4.7	1.99	2.24	2.2	4.62	21.94	18.99	5.75
**74**	5.35	14.86	10.54	1.41	5.58	3.23	4.54	2.71	4.91	20.41	16.18	5.4
**75**	7.28	26.57	17.83	1.45	7.22	5.37	6.86	7.14	9.56	21.46	18.93	6.4
**76**	11.37	35.72	24.44	2.22	18.06	7.96	8.66	8.77	19.92	22.47	19.64	9.33
**77**	9.02	28.62	21.59	1.58	11.98	6.51	6.75	7.5	10.91	25.59	20.12	7.81
**78**	7.75	24.99	19.73	2.63	9.18	5.64	6.99	7.37	8.78	22.56	19.5	7.18
**79**	5.79	19	11.95	1.05	4.49	3.81	4.98	6.09	3.73	20.57	13.82	4.95
**80**	6.44	22.15	15.86	1.49	6.78	4.61	6	6.75	6	20.01	16.26	5.74
**81**	5.32	32.53	25.4	1.2	6.63	3.46	4.76	3.78	6.26	46.71	21.86	5.67
**82**	6.66	23.46	13.94	1.05	3.46	4.9	6.59	6.24	4.01	23.69	19.83	5.05

**Table 4:** Cytotoxicity of compounds **47**–**50**, **53**, **54**, **57**, **59**, **60**–**63**, **65**–**70**, and **72** on cell cycle in CCRF-CEM lymphoblasts.

		% of Total Cellular Populations							
No	Conc. (µM)	Sub-G1 Apoptosis	G0/G1	S	G2/M	>G2/M	M ^a^	DNA ^b^ Synthesis	RNA ^b^ Synthesis
**47**	2.74 13.7	3.36 7.59	37.93 27.73	43.41 39.33	18.66 32.94	9.71 10.56	1.87 8.29	2.76 24.01	19.4 24.95
**48**	1.42 7.1	2.83 4.37	34.46 49.43	46.76 23.5	18.78 27.08	9.95 12.21	2.11 2.47	8.1 6.21	16.56 16.73
**49**	1.34 6.7	3.14 5.14	35.52 40.24	43.74 36.15	20.74 23.61	13.16 10.02	2.09 3.35	18.2 5.93	19.14 17.12
**50**	1.32 6.6	5.09 5.83	35.7 49.76	47.92 30.92	16.38 19.32	14.2 9.11	1.42 2.12	39.66 15.47	26.23 20.85
**53**	1.31 6.55	4.52 6.93	33.63 39.14	47.13 42.84	19.24 18.01	10.22 7.45	1.74 1.57	39.73 26.21	57 27.33
**54**	1.09 5.45	5.68 7.28	35.54 50.08	43.61 31.69	20.85 18.23	9.52 7.91	2.02 3.21	29.33 20.4	28.71 17.96
**57**	1.51 7.55	4.62 5.7	36 43	37.02 30.14	26.98 26.85	13.61 12.5	1.78 2.12	18.86 19.87	10.43 9.22
**59**	1.2 6	8.8 9.63	34.06 42.51	47.12 33.63	18.82 23.85	10.37 10.89	1.52 5.65	41.37 10.43	47.78 34.65
**60**	1.13 5.65	4.28 6.97	34.48 41.01	46.3 31.97	19.23 27.02	11.73 11.18	2.63 4.67	32.46 15.1	26.93 11.19
**61**	1.05 5.25	3.69 10.05	33.19 32.98	47.6 44.88	19.21 22.14	11.24 12.98	2.03 2.24	34.82 9.05	29.57 7.41
**62**	1.17 5.85	13.92 8.12	27.68 37.23	44.18 43.17	28.15 19.61	9.65 8.22	1.38 1.5	32.88 17.61	10.51 30.99
**63**	0.77 3.85	3.41 12.97	42.76 34.13	40.42 47.99	16.81 17.88	8.56 11.38	1.66 1.53	41.06 13.54	35.01 2.79
**65**	0.55 2.75	17.77 8.67	40.82 47.21	39.14 32.8	20.04 19.99	7.55 7.13	1 1.66	36.82 19.73	36.04 24.95
**66**	1.11 5.55	2.95 11.72	39.53 48.53	4387 24.9	16.6 26.56	10.42 10.45	1.64 2.29	39.87 9.68	29.44 3.05
**67**	1.05 5.25	3.13 11.06	37.85 40.09	43.93 40.3	18.22 19.61	9.56 8.98	1.59 2.27	42.05 9.76	38.15 6.99
**68**	1.23 6.15	2.71 7.16	35.73 48.5	45.73 31.86	18.54 19.64	9.87 9.65	1.73 2.11	41.27 7.18	37.4 3.8
**69**	1.38 6.9	3.3 13.89	43.48 38.16	35.7 42.58	20.83 19.27	8.58 12.52	1.61 1.6	34.45 4.22	31.54 2.64
**70**	1.16 5.8	3.21 13.75	39.54 33.44	38.46 43.31	21.99 23.25	10.54 10.33	1.57 1.08	35.58 4.88	35.28 2.88
**72**	2.14 10.7	4.05 10.12	47.22 31.36	34.32 46.65	18.46 21.98	8.35 13.12	1.61 5.28	27.07 4.35	21.64 2.19
**Control**	0	2.91	36.69	44.89	18.42	9.38	1.68	36.52	32.97

^a^ Mitosis Phospho-Histone3 (Ser10). ^b^ DNA and RNA synthesis in CCRF-CEM lymphoblasts (% positive of cells). Flow cytometry analysis was used to quantify cell cycle distribution and percentage of apoptotic cells. The sum of the percentage for G0/G1, S, and G2/M is equal to 100%.

## Data Availability

Data is contained within the article.
